# Cross-Platform Synaptic Network Analysis of Human Entorhinal Cortex Identifies TWF2 as a Modulator of Dendritic Spine Length

**DOI:** 10.1523/JNEUROSCI.2102-22.2023

**Published:** 2023-05-17

**Authors:** Courtney K. Walker, Kelsey M. Greathouse, Jennifer J. Tuscher, Eric B. Dammer, Audrey J. Weber, Evan Liu, Kendall A. Curtis, Benjamin D. Boros, Cameron D. Freeman, Jung Vin Seo, Raksha Ramdas, Cheyenne Hurst, Duc M. Duong, Marla Gearing, Charles F. Murchison, Jeremy J. Day, Nicholas T. Seyfried, Jeremy H. Herskowitz

**Affiliations:** ^1^Department of Neurology, Center for Neurodegeneration and Experimental Therapeutics, University of Alabama at Birmingham, Birmingham, Alabama 35294; ^2^Department of Neurobiology, University of Alabama at Birmingham, Birmingham, Alabama 35294; ^3^Department of Biochemistry, Emory University School of Medicine, Atlanta, Georgia 30322; ^4^Department of Pathology and Laboratory Medicine and Department of Neurology, Emory University School of Medicine, Atlanta, Georgia 30322

**Keywords:** Alzheimer's disease, dendritic spines, entorhinal cortex, proteomics, synapse, systems biology

## Abstract

Proteomic studies using postmortem human brain tissue samples have yielded robust assessments of the aging and neurodegenerative disease(s) proteomes. While these analyses provide lists of molecular alterations in human conditions, like Alzheimer's disease (AD), identifying individual proteins that affect biological processes remains a challenge. To complicate matters, protein targets may be highly understudied and have limited information on their function. To address these hurdles, we sought to establish a blueprint to aid selection and functional validation of targets from proteomic datasets. A cross-platform pipeline was engineered to focus on synaptic processes in the entorhinal cortex (EC) of human patients, including controls, preclinical AD, and AD cases. Label-free quantification mass spectrometry (MS) data (*n* = 2260 proteins) was generated on synaptosome fractionated tissue from Brodmann area 28 (BA28; *n* = 58 samples). In parallel, dendritic spine density and morphology was measured in the same individuals. Weighted gene co-expression network analysis was used to construct a network of protein co-expression modules that were correlated with dendritic spine metrics. Module-trait correlations were used to guide unbiased selection of Twinfilin-2 (TWF2), which was the top hub protein of a module that positively correlated with thin spine length. Using CRISPR-dCas9 activation strategies, we demonstrated that boosting endogenous TWF2 protein levels in primary hippocampal neurons increased thin spine length, thus providing experimental validation for the human network analysis. Collectively, this study describes alterations in dendritic spine density and morphology as well as synaptic proteins and phosphorylated tau from the entorhinal cortex of preclinical and advanced stage AD patients.

**SIGNIFICANCE STATEMENT** Proteomic studies can yield vast lists of molecules that are altered under various experimental or disease conditions. Here, we provide a blueprint to facilitate mechanistic validation of protein targets from human brain proteomic datasets. We conducted a proteomic analysis of human entorhinal cortex (EC) samples spanning cognitively normal and Alzheimer's disease (AD) cases with a comparison of dendritic spine morphology in the same samples. Network integration of proteomics with dendritic spine measurements allowed for unbiased discovery of Twinfilin-2 (TWF2) as a regulator of dendritic spine length. A proof-of-concept experiment in cultured neurons demonstrated that altering Twinfilin-2 protein level induced corresponding changes in dendritic spine length, thus providing experimental validation for the computational framework.

## Introduction

Mass spectrometry (MS)-based studies using postmortem human brain tissue samples have yielded robust assessments of the aging and Alzheimer's disease (AD) proteomes (Seyfried et al., 2017; Yu et al., 2018; McKetney et al., 2019; Bai et al., 2020; Johnson et al., 2020; Wingo et al., 2020). A prior advancement in the use of proteomic data were the generation of networks of protein co-expression modules, which could be correlated to other data, such as AD neuropathology or antemortem clinical memory scores (Cerami et al., 2010; Barabási et al., 2011; Tran et al., 2011; Clarke et al., 2013; Huan et al., 2013, 2015; Seyfried et al., 2017; Johnson et al., 2020). Hub proteins of modules that correlate with relevant disease traits have been nominated as candidates for drug or biomarker development (He et al., 2017; Sun et al., 2017; Toledo et al., 2017; Yue et al., 2017), making this a promising approach for making unbiased identification of proteins that regulate traits supplied to the network.

Most brain proteomic studies are performed on bulk homogenates of tissue, but this could potentially occlude specific proteomic signatures or alterations in subcellular compartments, such as synapses (Carlyle et al., 2018, 2021). Proteomic studies have been performed on fractions enriched for synapses from postmortem human tissue of AD patients and cognitively normal individuals (Chang et al., 2013; Zhou et al., 2013; Hesse et al., 2019; Carlyle et al., 2021). These studies identified AD-related changes in pathways including mitochondrial function, proteasome function, and synaptic function, all of which have been associated with AD pathogenesis (Bonet-Costa et al., 2016; Guo et al., 2017; Swerdlow, 2018). Thus, synapse-enriched fractions can be used to identify disease-relevant alterations of the proteome at the level of the synapse.

Dendritic spines house the postsynaptic compartment of excitatory synapses. These actin-based structures are highly plastic and change their size and shape in relation to synaptic activity and strength (Kasai et al., 2003; Matsuzaki et al., 2001). Such changes in dendritic spine size and shape depend on actin remodeling and are critical for long-term potentiation (LTP; Fukazawa et al., 2003; Bosch et al., 2014). Actin remodeling is a complex process that requires five major steps: actin monomer binding and transport, filament nucleation, filament assembly, capping, and actin severing (Lei et al., 2016). While some of these steps have been well studied with regard to dendritic spines, such as nucleation of filaments, less is known about other steps like actin monomer binding and transport.

Dendritic spines are affected in neurologic diseases, including autism spectrum disorders, AD, and schizophrenia. In AD, dendritic spines are lost in the dorsolateral prefrontal cortex (DLPFC; Boros et al., 2017), and synapse or dendritic spine loss is more strongly correlated with cognitive impairment than the hallmark pathologies of AD (DeKosky and Scheff, 1990; Terry et al., 1991; Boros et al., 2017; Chen et al., 2018). DLPFC dendritic spines can undergo structural remodeling in AD progression, including spine elongation with cognitive preservation or enlargement of head diameter with declining memory performance (Boros et al., 2017, 2019). Thus, proper regulation of dendritic spine structure is critical in both physiological and pathologic states.

The purpose of this study was to establish a pipeline that would facilitate greater contextualization of proteomic targets for more streamlined functional validation. To reach this goal, we focused on human synaptic processes and tested whether integration of proteomics with dendritic spine metrics could guide unbiased identification of a target protein that is functionally involved in regulating dendritic spines.

## Materials and Methods

### Human subjects

Postmortem samples of Brodmann area 28 (BA28) entorhinal cortex (EC) were obtained from subjects exhibiting a range of Alzheimer's disease (AD) pathology. Tissue samples were collected at the Emory University Alzheimer's Disease Research Center (ADRC). Cases were categorized into 3 diagnostic groups, which included (1) 22 cognitively normal controls without AD pathology, (2) 9 cognitively normal subjects with moderate to severe AD pathology at autopsy (CAD), and (3) 33 definite AD cases. Subsets of these cases were used for dendritic spine analysis or biochemical and proteomic analyses, as indicated in Tables 1 and 2. Samples of BA28 EC from the Emory ADRC were quite limited. As a result, the groups were not able to be matched for age or postmortem interval (PMI), with the exception of the cases used for biochemical analysis, which are matched for PMI. Additionally, very little tissue was available from cognitively normal individuals with AD pathology, resulting in the small sample size for that group.

**Table 1.** Case demographics

Case	Race/sex	Age at death	PMI (h)	ApoE	MMSE	Experiments
Control (*n* = 22)						
1	AF	57	17	E3/3	-	S T P
2	WM	40	31	E3/4	-	S T P
3	AM	59	6	E2/3	-	S T P
4	WF	52	3	E3/4	-	S T P
5	WM	57	10	E3/3	-	S T P
6	WF	46	6.5	E3/3	-	S T P
7	AM	53	6.5	E4/4	-	S T P
8	WF	78	12	E3/3	30	S T P
9	WF	92	16	E3/3	-	-- T P
10	WF	45	28	E3/3	-	S T P
11	WM	94	5.5	E3/3	29	S T P
12	AF	43	15	E3/3	-	S T P
13	WF	91	6	E3/3	-	S T P
14	WM	56	13	-	-	S T --
15	AF	61	6	-	-	S T P
16	AM	70	2.5	E3/3	29	S T P
17	WM	51	23	E2/3	-	S -- --
18	WF	75	6	E3/3	-	S -- --
19	WM	69	6	E3/3	-	S T P
20	AM	61	12	E3/4	-	S T P
21	WF	74	7	E3/3	-	S -- P
22	WF	74	3	E3/3	-	-- T P
CAD (*n* = 9)						
1	WM	76	36	E2/4	29	S T P
2	WF	64	17	E4/4	30	S -- --
3	WM	81	20	E3/3	27	S T P
4	WF	82	38	E3/4	-	S -- --
5	WM	89	19	E3/3	27	S T P
6	WM	80	5.5	E3/4	28	S T P
7	WM	87	21	E3/4	27	S T P
8	WF	87	5	E2/3	-	S T P
9	WF	96	12	E3/3	-	-- T P
AD (*n* = 33)						
1	WM	64	9	E3/4	-	S T P
2	WF	87	6	E3/4	-	S T P
3	WF	72	7	E3/4	-	S T P
4	WF	64	4.5	E3/4	-	S T P
5	AF	86	6	E3/3	15	S T P
6	WM	71	15	E3/4	-	S T P
7	WM	86	22	E3/4	-	S T P
8	WM	67	6.5	E2/3	-	S T P
9	WF	93	5	E3/4	6	-- T P
10	WM	85	4	E3/4	13	S -- --
11	WM	91	22	E3/3	-	S T P
12	WM	77	12	E3/4	-	S T P
13	WF	94	4	E3/4	19	S T P
14	WM	77	28	E3/4	0	-- T P
15	WM	94	40	E3/4	18	-- T P
16	HM	76	21	E3/3	15	S T P
17	WM	83	5.5	E3/4	10	S T P
18	WM	67	18	E3/3	-	S T P
19	AF	66	16	E3/4	-	S T P
20	WM	74	2.5	E3/3	6	S T P
21	WM	62	6	E2/3	-	-- T P
22	WM	68	15	E3/3	-	S T P
23	WF	88	5	E3/4	0	-- T P
24	WM	80	4	E3/4	23	S T P
25	AM	86	7	E4/4	12	-- T P
26	WF	80	6	E3/4	-	-- T P
27	AF	80	5	E3/4	-	-- T P
28	WF	91	5	E3/4	-	S -- --
29	WM	79	6	E4/4	-	S T P
30	F	85	9.5	E4/4	-	S T P
31	WF	81	12	E3/4	0	S T P
32	WF	75	12	E4/4	-	S T P
33	WF	93	16	E3/4	-	-- T P

CAD = cognitively normal individuals with Alzheimer's disease pathology. A = African American. H = Hispanic. W = White. F = Female. M = Male. MMSE = Mini Mental State Examination. S = Spine Morphometry. T = Tau Quantification. P = Proteomics.

**Table 2.** Case pathology data

Case	Frontal NP	Frontal DP	CERAD score	Frontal NFT	Braak stage	ABC score	Experiments
Control (*n* = 22)							
1	-	-	-	-	I	-	S T P
2	None	Moderate	-	None	0	-	S T P
3	None	None	-	None	I	None	S T P
4	None	Frequent	B	None	0	Low	S T P
5	-	-	-	-	II	-	S T P
6	None	None	-	None	0	-	S T P
7	-	-	-	-	II	-	S T P
8	None	None	-	None	II	None	S T P
9	-	-	-	-	III	-	-- T P
10	-	-	-	-	0	-	S T P
11	None	None	-	None	II	-	S T P
12	-	-	-	-	0	-	S T P
13	-	-	A	-	III	Low	S T P
14	None	None	-	None	I	-	-- T P
15	None	Sparse	-	None	II	-	S T P
16	None	Moderate	-	None	I	None	S T P
17	-	-	-	-	-	-	S -- --
18	None	None	0	None	I	-	S -- --
19	-	-	-	-	II	-	S T P
20	None	Moderate	-	None	II	-	S T P
21	-	-	-	-	-	-	S -- P
22	-	-	-	-	I	-	-- T P
CAD (*n* = 9)							
1	Frequent	Moderate	C	None	IV	-	S T P
2	Frequent	Frequent	C	Sparse	II	Low	S -- --
3	Sparse	Moderate	B	None	II	Low	S T P
4	Frequent	Frequent	C	None	III	Intermediate	S -- --
5	Frequent	Frequent	C	None	IV	Intermediate	S T P
6	Frequent	Frequent	C	Sparse	IV	Intermediate	S T P
7	Moderate	None	B	Sparse	I	Intermediate	S T P
8	Frequent	Frequent	C	Sparse	III	Intermediate	S T P
9	-	-	-	-	IV	-	-- T P
AD (*n* = 33)							
1	Frequent	None	C	Frequent	V-VI	High	S T P
2	-	-	-	-	V-VI	-	S T P
3	Frequent	Frequent	C	Moderate	VI	High	S T P
4	-	-	-	-	VI	-	S T P
5	Frequent	Moderate	C	None	II	Low	S T P
6	-	-	-	-	IV	-	S T P
7	-	-	-	-	VI	-	S T P
8	-	-	-	-	VI	-	S T P
9	Frequent	Moderate	C	Frequent	VI	High	-- T P
10	Frequent	Frequent	C	Frequent	VI	High	S -- --
11	-	-	-	-	V	-	S T P
12	Frequent	Frequent	C	Frequent	VI	High	S T P
13	Moderate	Frequent	C	Sparse	IV	Intermediate	S T P
14	Frequent	Frequent	C	Frequent	IV	Intermediate	-- T P
15	Frequent	Frequent	C	Sparse	I	Low	-- T P
16	Frequent	Moderate	C	Sparse	IV	Intermediate	S T P
17	Frequent	Sparse	C	Frequent	V	High	S T P
18	-	-	-	-	VI	-	S T P
19	-	-	-	-	VI	-	S T P
20	Frequent	Frequent	C	Moderate	VI	High	S T P
21	-	-	-	-	VI	-	-- T P
22	-	-	-	-	VI	-	S T P
23	Frequent	Moderate	C	Sparse	IV	Intermediate	-- T P
24	Frequent	Frequent	C	Sparse	IV	Intermediate	S T P
25	Sparse	Frequent	C	Sparse	IV	Intermediate	-- T P
26	-	-	-	-	IV	-	-- T P
27	-	-	-	-	VI	-	-- T P
28	Frequent	Frequent	C	Frequent	-	High	S -- --
29	-	-	-	-	-	-	S T P
30	Frequent	Moderate	C	Moderate	VI	High	S T P
31	Frequent	None	C	Frequent	VI	High	S T P
32	-	-	-	-	VI	-	S T P
33	Frequent	Frequent	C	None	III	Intermediate	-- T P

CAD = cognitively normal individuals with Alzheimer's disease pathology. NP = neuritic plaque. DP = diffuse plaque. CERAD = Consortium to Establish a Registry for Alzheimer's Disease. NFT = neurofibrillary tangle. ABC = Amyloid Braak CERAD score. S = spine morphometry. T = tau quantification. P = proteomics.

The case diagnosis is based on Mini-Mental State Examination (MMSE), Consortium to Establish a Registry for Alzheimer's Disease (CERAD) criteria for the neuropathologic diagnosis of Alzheimer's disease, and Braak staging of neurofibrillary tau pathology. Although multiple neuropsychological tests were employed in the cognitive testing of these subjects, the MMSE is the most commonly used test for complaints of memory problems or when a diagnosis of dementia is being considered, and those results are presented in Table 1. The MMSE score ranges from 0 to 30, with 30 indicating no cognitive impairment. CAD cases have an MMSE score no lower than 27, and severe to moderate AD patients have MMSE scores of 10–20 out of 30. At the end stages of disease, impairment is severe enough to prevent testing. Pathology data on cases is presented in Table 2. Neuritic and diffuse plaques were scored semiquantitatively according to CERAD methods (0, A–C, or none, sparse, moderate, frequent; Mirra et al., 1991). CAD cases have moderate to frequent plaques based on the CERAD score. For the AD cases with available pathology data, all cases had frequent plaques. Tau neurofibrillary tangle (NFT) accumulation was scored via Braak staging (0–VI). CAD cases had neurofibrillary tangle accumulation ranging from Braak stage I–IV. The AD cases ranged from Braak stages I-VI, although the vast majority were within the IV–VI range. The Amyloid Braak CERAD (ABC) score (none, low, intermediate, high) was used as a global measure of AD pathology (Montine et al., 2012). CAD cases ranged from low to intermediate on the ABC scale. AD cases ranged from low to high on the ABC scale, but the majority of cases were in the intermediate to high range. It is important to note that the majority of these cases had no coexisting pathologies, such as stroke or Lewy body disease. The ApoE Risk score was determined for each individual by assigning each ε2 allele as −1, ε3 as 0, and ε4 as +1, based on the protective, neutral, and detrimental effects of each allele on AD risk. The two numbers, one for each allele, are added to yield the ApoE Risk for each individual, with the possible values ranging from −2 (ε2/ε2 genotype; not present in our cohort) to +2 (ε4/ε4 genotype).

#### Synaptosomal fractionation

Frozen human entorhinal cortex samples were used for subcellular fractionation. Synaptosomal fractionation was performed based on previously described methods (Greathouse et al., 2018; Hallett et al., 2008). Two small sections from each frozen entorhinal cortex sample were obtained from opposite ends of the sample with a razor blade and were thawed separately in 75 µL of ice cold TEVP buffer + 320 mm sucrose, and homogenized. The initial homogenate was centrifuged at 800 × *g* for 10 min at 4°C. The resulting pellet was resuspended in 8 m urea buffer and saved as the insoluble fraction. A total of 30 µl of the supernatant was saved as the whole homogenate, and the remainder was centrifuged at 9200 × *g* for 15 min at 4°C. The resulting supernatant was saved, and the pellet was resuspended in 60 µl of TEVP buffer + 35.6 mm sucrose, vortexed, and left on ice for 30 min. Next, the resuspended pellet was centrifuged at 21,130 × *g* for 30 min at 4°C. The resulting supernatant was saved, and the pellet was rinsed in 100 µl of TEVP buffer (10 mm Tris base, 1 mm Na_3_VO_4_, 5 mm NaF, 1 mm EDTA, 1 mm EGTA, pH 7.4) and resuspended in 30 µl of TEVP buffer, resulting in the synaptosomal fraction. All obtained fractions were stored at −80°C. Protein concentrations for synaptosome and insoluble fractions were determined by Pierce bicinchoninic acid (BCA) protein assay (catalog #23227, Thermo Scientific) before use.

#### Western blotting

Samples were added to loading dye to yield the desired amount of protein in a final volume of 15–20 µl. The samples were then boiled at 95°C for 4 min, spun down briefly in a benchtop centrifuge, then loaded on either precast 4–15% gradient gels (catalog #5671085, Bio-Rad) or hand-cast 10% polyacrylamide gels. Blots were run for 20 min at 50 V, then 40–45 min at 200 V. Samples were then transferred from the polyacrylamide gel to a polyvinylidene fluoride membrane (catalog #1620264, Bio-Rad) at 0.10 A for 16 h. Membranes were blocked in 1× Casein Blocking Buffer (catalog #B6429, Sigma-Aldrich) at room temperature for 1 h, then incubated in primary antibody overnight at 4°C. The next day, membranes were washed in 1× TBS + 0.1% Tween for 5 min at room temperature, followed by two 5-min washes in 1× TBS. Membranes were then incubated in secondary antibody for 1 h at room temperature, followed by another set of washes. Images were captured on an Odyssey Image Station (Li-Cor). For relative protein quantification, band intensities were quantified using the Odyssey Application Software (Li-Cor, version 3.0).

Primary antibodies: PSD95 rabbit monoclonal (catalog #3450, Cell Signaling Technology, RRID:AB_2292883), synaptophysin rabbit monoclonal (catalog #5461, Cell Signaling Technology, RRID:AB_10698743), GAPDH mouse monoclonal (catalog #MAB374, MilliporeSigma, RRID:AB_2107445), anti-human tau rabbit polyclonal (catalog #A0024, Agilent, RRID:AB_10013724), TWF2 rabbit polyclonal (catalog #NBP2-47591, Novus Biologicals, RRID:AB_2895596). Secondary antibodies: IRDye 800CW goat anti-mouse IgG secondary antibody (catalog #926-32210, LI-COR Biosciences, RRID:AB_621842), goat anti-rabbit IgG (H+L) highly cross-adsorbed secondary antibody, Alexa Fluor 680 (catalog #A-21109, Thermo Fisher Scientific, RRID:AB_2535758).

#### Electron microscopy

Synaptosome pellets were fixed in EM GRADE ULTRAPURE fix 3% glutaraldehyde in 0.1 m sodium cacodylate buffer pH 7.2. After fixation the specimens are rinsed several times with sodium cacodylate buffer pH 7.4 0.1 m followed by postfixation with 1% osmium tetroxide in cacodylate buffer for 1 h in the dark. After rinsing again with Cacodylate buffer for four changes at 15 min each, the specimens are dehydrated through a series of graded ethyl alcohols from 50% to 100%. The schedule is as follows: 50% for 5 min., 2% uranyl acetate in 50% EtOH for 30 min in dark, 50% for 5 min, 80% for 5 min, 95% for 5 min, and four changes of 100% for 15 min each. After dehydration the infiltration process requires steps through an intermediate solvent, 2 changes of 100% propylene oxide (P.O.) for 10 min each and finally into a 50:50 mixture of P.O. and the embedding resin (Embed 812, Electron Microscopy Sciences) for 12–18 h. The specimen is transferred to fresh 100% embedding media. The following day, the specimen is then embedded in a fresh change of 100% embedding media. Blocks polymerize overnight in a 60°C embedding oven and are then ready to section. The resin blocks are first thick sectioned at 0.5 μm with a histo diamond knife using an Ultracut UCT 7 (Leica) sections are collected on slides and stained with Toluidine Blue. These sections are used as a reference to trim blocks for thin sectioning. The appropriate blocks are then thin sectioned using a diamond knife (Diatome, Electron Microscopy Sciences) at 70–90 nm (silver to pale gold using color interference), and sections are then placed on copper grids (Electron Microscopy Sciences). After drying, the sections are stained with the heavy metals uranyl acetate and lead citrate for contrast. After drying, the grids are then viewed on a Tecnai Spirit 120kv TEM (FEI). Digital images are taken with an AMT BioSprint 29 camera (AMT Inc.).

#### ELISAs

ELISA kits were used to detect levels of total human tau (catalog #KHB0041, Invitrogen) and human tau phosphorylated at S199 (catalog #KHB7041, Invitrogen), T231 (catalog #KHB8051, Invitrogen), and S396 (catalog #KHB7031, Invitrogen). For synaptosomal total tau, samples were diluted 1:20 (1 µg protein per well) and the calculated concentration was multiplied by 20 to account for the dilution. For insoluble total tau, samples were diluted 1:1000 (0.02 µg protein per well) and the calculated concentration was multiplied by 1000 to account for the dilution. For all phospho-tau ELISAs, 20 µg of protein were loaded per well. ELISAs were read at 450 nm on a VersaMax microplate reader (Molecular Devices).

#### Synaptosome proteomics

##### Liquid chromatography coupled to tandem mass spectrometry

Protein concentrations for the 58 individual synaptic fractions were determined by BCA assay, and 100 µg of total protein was aliquoted for each sample. Synaptosome fractions were treated with 1 mm (final concentration) dithiothreitol (DTT) at 25°C for 30 min, followed by 5 mm (final concentration) iodoacetimide (IAA) at 25°C for 30 min in the dark and digested with 1:100 (w/w) lysyl endopeptidase (Wako) at 25°C overnight. The samples were then diluted 2-fold with 50 mm NH_4_HCO_3_ and further digested overnight with 1:50 (w/w) trypsin (Promega) at 25°C. The samples were then acidified and desalted with a Sep-Pak C18 column (Waters). All samples were dried under vacuum. The dried peptides were resuspended in peptide loading buffer (0.1% formic acid, 0.03% trifluoroacetic acid, 1% acetonitrile). Peptide pools representing an equivalent amount of peptides from all cases were also created as reference standards for subsequent liquid chromatography coupled to tandem mass spectrometry (LC-MS/MS) analysis. Peptide mixtures (1 µg) were separated on a self-packed C18 (1.9 µm) fused silica column (25 cm × 75 μm internal diameter; New Objective) by Thermo Easy-nLc 1200 and monitored on an Orbitrap Fusion Lumos mass spectrometer (Thermo Fisher Scientific). Elution was performed over a 100 min gradient at a rate of 250nL/min with buffer B ranging from 3% to 35% (buffer A: 0.1% formic acid in water, buffer B: 0.1% formic in 80% acetonitrile). The mass spectrometer cycle was programmed to collect in top speed mode where each cycle consists of one full MS scan followed by however many data-dependent MS/MS scans that can fit within 3 s. The MS scans (300–1500 m/z range, 400,000 AGC, 500-ms maximum ion time) were collected at a resolution of 120,000 at m/z 200 in profile mode and the MS/MS spectra (1.5 m/z isolation width, 30% collision energy, 50,000 AGC target, 22 ms maximum ion time) were acquired at a resolution of 7500 at m/z 200. Dynamic exclusion was set to exclude previous sequenced precursor ions for 10 s within a 10-ppm window. The 58 samples were randomized into three batches with the pooled standards analyzed in duplicate at the beginning and end of each batch (*n* = 8 replicates). All raw files are uploaded onto Synapse at https://www.synapse.org/DendriteStudy.

##### Weighted protein co-expression network analysis

Before network analysis, the 66 single-shot LFQ samples (58 plus eight pooled external standards, labeled “GIS”) were subjected to the previously published median polish of ratio over central tendency (TAMPOR) for correction of batch effects across the three batches (Johnson et al., 2020). Protein rows with <50% missing values were retained, and then successive iterations of median polish leverage the central tendency of both the GIS and all non-GIS samples randomized within each batch, for each protein, driving the medians within batch toward the central tendency across batches, without reducing or introducing intrabatch variance. Output after 75 iterations was expressed as log2(LFQ/central tendency within row), and all columns have a median log_2_ ratio of zero because of the two-way table median polish implementation. One sample with low network connectivity Z.K < 2.5 SDs (outlier samples) was identified in an iterative removal and check process using the SampleNetworks R package (version 1.06). Bootstrap regression of the LFQ intensity matrix using a model incorporating case status and case covariates for age, sex and PMI was performed. A weighted protein co-expression network was built using the above LFQ-normalized, outlier-removed, postregressed protein expression values using the WGCNA blockwiseModules function with the following parameters: soft threshold power β = 9.5, deepSplit = 4, minimum module size = 14, merge cut height = 0.07, signed network with partitioning about medioids respecting the dendrogram, minimum kME to stay of 0.30, TOMDenom parameter set to “mean,” and a reassignment threshold of *p* = 0.05. The resulting modules were used to calculate module eigenproteins (MEs; the 1st principal component of the module). MEs were correlated with biological traits including case demographic and pathology data, dendritic spine density and morphology, and synaptosomal and insoluble phospho-tau levels.

##### Gene ontology enrichment analysis

Gene ontology (GO)-Elite (version 1.2.6) was run through AltAnalyze (version 2.1.4.3), using Ensembl as the reference database for the human proteome. Ontology terms were pruned using the *z* score and a Fisher exact test was used for overrepresentation analysis (ORA). The *z* score cutoff for initial filtering was set at 1.96, and the permuted p-value cutoff was set to 0.05. The minimum number of changed genes was limited to 5 and the number of permutations for ORA = 2000.

### Human tissue processing and Golgi–Cox staining

Human tissue samples were obtained from the Emory Alzheimer's Disease Research Center. All tissue samples were fixed in 4% paraformaldehyde (PFA) immediately following dissection and stored in preservative solution containing sodium azide at 4°C. Tissue blocks of ∼20 × 20 × 5 mm taken from the entorhinal cortex [Brodmann area 28 (BA28)] were sectioned into 250-µm slices (∼15 per block) using a Leica vibratome (VT1000S, Leica Biosystems) and stored in preservative solution (0.1% wt/vol sodium azide in phosphate-buffered saline) until Golgi–Cox impregnation. All tissues were stained using the FD Rapid Golgi Stain kit (catalog #PK401, FD Neurotechnologies), following the manufacturer's instructions with the following modifications. Tissue slices were impregnated in chromate mixture of Solution A (potassium dichromate and mercuric chloride) and Solution B (potassium chromate). The chromate solution was replaced after the first 24 h, and tissue was then left in chromate solution in the dark for six weeks. Next, tissue slices were immersed in Solution C for 48 h, and this solution was replaced after 24 h, according to manufacturer's instructions. Tissues were then plated on 75 × 25 mm gelatin-coated slides (catalog #PO101, FD Neurotechnologies) using additional Solution C and allowed to dry in the dark for 2 h. Next, tissues were submerged sequentially in mixtures of Solution D, Solution E, and distilled water according to the manufacturer's instructions. After rinsing with distilled water, tissues were dehydrated with graded alcohols (70%, 90%, and 100% ethanol in deionized water) and cleared with xylenes (catalog #X3P-1GAL, Thermo Fisher Scientific). Slides were sealed with Permount Toluene Solution (catalog #SP15-100, Fisher Chemicals) and coverslipped with spacers (Secure Seal Spacer, 20-mm diameter × 0.12-mm depth, catalog #70327-205, Electron Microscopy Sciences) and 50 × 24-mm glass (cover glass, rectangles, 24 × 50 mm, thickness = 0.13–0.17 mm, catalog #633153, Carolina Biological). Slides were stored in opaque slide holders (catalog #12-587-10, Fisher Scientific) at room temperature.

### Dendrite imaging/brightfield microscopy

Dendrites on pyramidal neurons in layers 2 and three of BA28 entorhinal cortex were imaged. For each case, many tissue slices were Golgi stained. From each tissue slice, 2 or more cells were imaged and analyzed. 10–20 Golgi-stained cells were sampled per case. Each slide contained multiple slices of Golgi-stained tissues from the same original block of patient tissue. A minimum of two or more neurons were imaged from each slice of tissue. The amount of slices imaged per slide was not constrained by PMI, but in the AD patient group, it was occasionally constrained because of the expected neuronal loss in AD. AD cases had fewer total Golgi-stained neurons, therefore more tissue slices were required to be powered. From each cell, a single dendritic segment was imaged. The following criteria were used to select cells for imaging: (1) located centrally within the tissue sample depth, (2) not obscured by large staining debris, and (3) fully impregnated. If the cell met the criteria, a single dendritic length was imaged. Dendrite selection criteria were: (1) unobstructed/isolated/not overlapping other dendrites, (2) length >30 µm, and (3) diameter ∼1 µm. If two or more dendrites fulfilled the criteria from a single cell, the first dendrite clockwise was the only dendrite selected. If no dendrites from a cell fulfilled the criteria, another cell was selected. All imaging was conducted by blinded experimenters. Each tissue slice was initially viewed under 10× magnification to establish the region of interest. Next, a dendrite within the region of interest was viewed at 60× magnification to determine whether the dendrite fulfilled the above criteria. Z-stacks were captured with a z-step size of 0.1 µm at 60× using a Nikon Plan Apo 60×/1.40 NA oil-immersion objective on a Nikon Eclipse Ni upright microscope with a lumen 200 light source and Nikon DS-43 Digital Sight for brightfield microscopy. Each image was captured using the following parameters: lamp, 100%; field stop, 1.5 mm; exposure, 60 ms; analog gain, 2.0–2.4×; image size, 1028 × 1028 pixels.

### Three-dimensional digital image reconstruction (Golgi)

Dendrite and dendritic spine reconstructions were conducted by blinded experimenters. Image stacks of neuronal dendrites were converted to 16-bit TIFF files in ImageJ, then imported to Neurolucida 360 (2.70.1, MBF Biosciences). First, images were assessed for quality. Those with dendritic spines that were difficult to discern were excluded from analysis. At random human neurons that are Golgi-stained can exhibit blebbing along dendritic branches that interfere with the ability to discern individual dendritic spines. If a dendrite exceeded 25 µm, as measured in the *z* plane, the dendrite and spines will exhibit z-smear, rendering it impossible to accurately identify boundaries of individual spines (Walker et al., 2022). The blinded experimenter would decide, after the image was in Neurolucida software, whether the dendrite and spines were suitable for analysis. PMI or group did not influence occasional poor image quality. Dendrites were traced using a semiautomated directional kernel algorithm. Spines were traced using voxel clustering. Initiation and termination points for dendrite reconstruction were established using the following criteria: (1) ≥10 µm away from the distal tip of the dendrite, (2) consistent diameter, (3) level axis with limited movement in the z plane, and (4) ≥30 µm in length. Next, the experimenters manually examined each assigned point in the *x*, *y*, and *z* planes to verify that the points were located on the dendrite and were not artificially assigned. The dendrite was first viewed at *x-z* or *y-z* planes to ensure that points were correctly positioned at the midline of the dendrite. Points were then verified in the *x*-*y* plane, and the diameter of the reconstruction at each point was confirmed to match the dendrite diameter. Dendritic spine reconstruction used the following parameters for classification: outer range, 7 µm; minimum height, 0.3 µm; detector sensitivity, 90–125%; minimum count, eight voxels. The morphology of each reconstructed spine was examined to verify that axial smear did not cause misrepresentation, and the merge and slice tools were used to correct inaccuracies. The position of each spine backbone point was confirmed by the experimenter. To correct a misrepresentative backbone, the spine was viewed from the *z* plane, and experimenters moved backbone points in the *x*-*y* plane. Any repositioning in the *x-z* or *y-z* plane was performed while the spine was being viewed from the lateral angle.

Morphometric analysis was conducted for each spine, and measurements categorized spines into thin, stubby, mushroom, and filopodia classes. For spine classification, the following established parameters were used: head-to-neck ratio, 1.1; length-to-head ratio, 2.5; mushroom head size, 0.35 µm; filopodium length, 3.0 µm. Spines with a head-to-neck ratio > 1.1 and head diameter >0.35 µm were classified as mushroom. Spines were classified as filopodia or thin if head-to-neck ratio was <1.1 and either (1) length-to-head ratio was >2.5 or (2) head size was <0.35 µm. Of these, if the total length was >3.0 µm, the spine was classified as filopodia, and if <3.0 µm, as thin.

Reconstructions were exported to Neurolucida Explorer (2.70.1, MBF Biosciences), where data were collected for quantitative analysis. The dendritic spine measurement parameters included spine length and spine head diameter, among others. These parameters were exported and collected in Microsoft Excel. Derived measurements, such as spine density, were calculated from raw measurement data. Spine density was calculated by determining the number of spines per 10 µm of dendrite length. Spine length was defined as the curvilinear backbone length from the insertion point to the most distal point of the spine head. Head diameter was defined as the breadth of the spine head at its widest cross-sectional point. Both morphologic measurements and corresponding backbone reconstructions were verified.

In total, 7446 µm of dendrite length and 5363 spines were analyzed in this study. Approximately 125 spines per control, 107 spines per CAD, and 83 spines per AD case were analyzed.

### CRISPR/dCas9 gRNA construction

Single guide RNAs (sgRNAs) for *Twf2* were designed using the online resource CHOPCHOP (http://chopchop.cbu.uib.no; Labun et al., 2019). Corresponding oligonucleotide sequences (Sigma Aldrich and Integrated DNA Technologies) with 5′ overhangs were inserted at *BbsI* cut sites into a lentiviral guide backbone containing an mCherry reporter (catalog #114199, Addgene) as previously described (Savell et al., 2019). Successful insertion of guides into the lentiviral backbone was confirmed by gel electrophoresis of products amplified using a forward primer for the U6 promoter and antisense primer specific to the inserted gRNA. A gRNA targeting the bacterial gene *lacZ* was used as a nontargeting control gRNA for all CRISPR/dCas9 experiments (Platt et al., 2014). All crRNA sequences were analyzed with NCBI's Basic Local Alignment Search Tool to ensure specificity. Enzymatically dead Cas9 (dCas9) CRISPR effector constructs containing a FLAG-tag and driven by the human synapsin 1 (hSyn) promoter were used in parallel with sgRNAs to alter target gene expression in neurons. For CRISPR activation (CRISPRa) experiments, dCas9 fused to transcriptional activator domain VPR (dCas9-VPR; catalog #114196, Addgene) was used for induction of gene expression.

### Cell lines

HEK93T cells (catalog #CRL-3216, ATCC, RRID:CVCL_0063) were used for lentivirus production. Cells were maintained in DMEM: high glucose, pyruvate (DMEM; catalog #11995073, Invitrogen) + 10% fetal bovine serum (catalog #35016CV, Corning) + 1% penicillin-streptomycin (catalog #15140122, Invitrogen) at 37°C with 5% CO_2_. HEK293T cells were split as needed.

### Primary neuron cultures

Rat hippocampal neurons were isolated from E18 Sprague Dawley rat embryos by digestion of hippocampal tissue with trypsin for 10 min, followed by sequential trituration (two fire-polished Pasteur pipettes of decreasing diameter). For validation of rat Twf2 CRISPRa guide RNAs, cells were cultured for immunocytochemistry (ICC), qPCR, and Western blotting. For ICC, cells were plated at a density of 4 × 10^5^ cells per coverslip on 18 mm glass coverslips (catalog #64-0714, Warner Instruments) coated with poly-L-lysine (catalog #P2636, Sigma-Aldrich; 1 mg/ml in borate buffer). For qPCR, cells were plated at a density of 1.25 × 10^5^ cells per well on a 24-well plate coated with poly-L-lysine (1 mg/ml in water). For Western blotting, cells were plated at a density of 4 × 10^5^ cells per well on a 12-well plate coated with poly-L-lysine. Cultures were maintained for 14 d *in vitro* (DIV) in a humidified incubator (5% CO_2_) at 37°C in Neurobasal medium supplemented with serum-free B27 and GlutaMAX (catalog #35050061, Invitrogen). Cultures received half media changes every 3 DIV until DIV14 when cells were processed for ICC, qPCR, or Western blotting.

For dendritic spine experiments, cells were cultured at a density of 4 × 10^5^ cells per coverslip on 18 mm glass coverslips coated with poly-L-lysine. Neurons were cultured in supplemented Neurobasal medium. Neurons were treated at DIV4 with 4 μm cytosine β-D-arabinofuranoside hydrochloride (catalog #C6645, Sigma-Aldrich) to eliminate the presence of native glial cells on the glass coverslips. Half-media changes occurred every 3 d with glia-conditioned supplemented Neurobasal medium.

### Lentivirus production and transduction

All lentivirus experiments were conducted under BSL-2 guidelines. Lentiviral constructs were produced by HEK293T cell transfection (catalog #CRL-3216, ATCC) with FugeneHD (catalog #E2312, Promega), OptiMEM (catalog #31985070, Life Technologies), packaging plasmid pxPAX2 (catalog #12260, Addgene), envelope plasmid pCMV-VSVG (catalog #8454, Addgene), custom guide RNAs (gRNAs) and CRISPR/dCas9 plasmids for 48 h. Media containing lentiviruses was harvested, passed through a 0.45 µm filter, and concentrated by centrifugation on an Optima L100K Ultracentrifuge at 25,000 rpm for 1 h and 45 min at 4°C. The viral pellet was resuspended in sterile 1× PBS, aliquoted into single-use tubes, and stored at −80°C until the day of transduction. Physical lentiviral titers (GC/ml) were determined using a Lenti-X qRT-PCR titration kit (catalog #631235, Takara Bio).

For transduction, on DIV4, half the media was removed from neuronal cultures and lentiviral vectors were transduced overnight at an MOI = 1000. On DIV5, virus was washed off with supplemented Neurobasal media and cells received a half media change. Successful transduction was confirmed on DIV14 with live cell imaging of mCherry signal and RT-qPCR.

### RNA extraction and qPCR

Total RNA was extracted from DIV14 primary hippocampal cultures to confirm gene expression changes using a commercially available kit (catalog #74106, QIAGEN). RNA was reverse-transcribed into cDNA using the iScript cDNA synthesis kit (catalog #1708841, Bio-Rad). cDNA was used for real-time qPCR verification of *Twf2* and the housekeeping gene, *Gapdh*.

### Oligonucleotides and primers

#### Oligonucleotides

Rat Twf2 sense: 5′-CACCGGGTCCGAATGTCTCAGTGC-3′. Rat Twf2 antisense: 5′-AAACGCACTGAGACATTCGGACCC-3′.

#### Primers

Rat Gapdh Forward: 5′-ACCTTTGATGCTGGGGCTGGC-3′. Rat Gapdh Reverse: 5′-GGGCTGAGTTGGGATGGGGACT-3′. Rat Twf2 Forward: 5′-ACTCAAGCAGAAGACGGTCAA-3′. Rat Twf2 Reverse: 5′-AACACCACAGATTCGAGGGA-3′. U6 Forward: 5′-TTTCTTGGGTAGTTTGCAGTTTT-3′.

#### Transfections

Primary rat hippocampal neurons were transfected on DIV12 using Lipofectamine 2000 transfection reagent (catalog #11668019, Invitrogen). Each well received 4.8 µg plasmid DNA (1.6 µg DNA per plasmid) and 2 µl Lipofectamine 2000 in unsupplemented Neurobasal medium. Neurons received a half-media change with glia-conditioned supplemented Neurobasal medium at 3 h 45 min post-transfection. Coverslips were fixed 48 h post-transfection.

#### ICC

Media were aspirated, and cells were fixed in room temperature 2% PFA at room temperature for 25 min. Coverslips were then washed four times in rinse buffer (50 ml 10× PBS, 2.5 ml normal goat serum, 2.5 ml normal horse serum, 0.25 g saponin in 500 ml dH_2_O), followed by permeabilization in blocking buffer (20 ml 10× PBS, 10 ml normal goat serum, 10 ml normal horse serum, 2 g bovine serum albumin, 0.1 g saponin in 500 ml dH_2_O) with 0.05% Triton X-100 for 30 min. The coverslips were then washed three times in rinse buffer, then incubated with anti-FLAG primary antibody (1:250, catalog #MAI-91878, Invitrogen, RRID: AB_1957945) at 4°C overnight. The next day, the coverslips were washed four times in rinse buffer, then incubated in Alexa 488 goat anti-mouse secondary antibody (1:100, catalog #A-10667, Invitrogen, RRID: AB_2534057) for 1 h at room temperature, protected from light. After secondary incubation, coverslips were washed three times in rinse buffer, with the third wash containing 4′,6-diamidino-2-phenylindole (DAPI) to stain nuclei. Coverslips were mounted using Vectashield mounting medium and sealed the next day with clear nail polish.

### Harvesting cells for Western blot analysis

Twelve-well culture plates were removed from the incubator and placed on ice. Media were aspirated from each well. One-milliliter 1× PBS with protease and phosphatase inhibitors was added to each well. Wells were scraped with a cell scraper and contents were transferred to a 1.5-ml tube. Tubes were centrifuged at 2300 rpm for 5 min at 4°C. The supernatant was discarded and pellets were resuspended in lysis buffer diluted to 1× in PBS with protease and phosphatase inhibitors. 5× lysis buffer: 0.5% Nonidet P-40, 0.5% deoxycholate, 150 mm sodium chloride, and 50 mm Tris, pH 7.4. Samples were vortexed and incubated on ice for 20 min. Samples were then centrifuged at 2300 rpm for 10 min at 4°C. The resulting supernatant was saved and stored at −80°C.

### Coverslip fixation

Coverslips were fixed 48 h post-transfection. Media were aspirated, and coverslips were incubated in room temperature 2% paraformaldehyde (PFA; catalog #P6148, Sigma-Aldrich) at room temperature for 20 min. Coverslips were washed in 1× PBS, followed by 1× PBS with 4′,6-diamidino-2-phenylindole (DAPI). Coverslips were mounted on glass slides with Vectashield mounting medium (catalog #H-1000, Vector Laboratories) and stored in the dark overnight at 4°C. The next day, excess Vectashield was removed and coverslips were sealed with clear nail polish. Slides were stored in the dark at 4°C.

### Widefield microscopy

While human proteomics and dendritic morphometry experiments were completed using entorhinal cortex samples, hippocampal cultures were used for *in vitro* experiments. Cells obtained from hippocampi from embryonic rats are predominantly pyramidal neurons with highly characteristic cell morphology and minimal interneurons and glia (Swanger et al., 2015; Henderson et al., 2019). In contrast, cortical cultures have much greater cell heterogeneity, deeming dendritic morphology experiments more challenging. Dendritic spine density and morphology were measured on pyramidal neurons in layers 2 and three of BA28 EC human samples. Therefore, in rat primary hippocampal neuron cultures, only pyramidal neurons were selected for image capture, using a Hamamatsu ORCA-flash 4.0 digital camera (catalog #C13440, Hamamatsu) on a Nikon Ti2 Eclipse widefield microscope. Samples were illuminated using a Lumencor sola light engine. For ICC, images were acquired at 20× using a 0.75 NA Plan Apo air objective, with the following parameters: z-step, 0.9 µm; image field, 1024 × 1024; FITC sola intensity, 20%; FITC exposure, 90 ms; TRITC sola intensity, 25%; TRITC exposure, 100 ms. Z-stacks of dendrites on DIV14 rat primary hippocampal neurons were acquired at 60× using a 1.5 NA oil-immersion objective, with the following parameters: z-step, 0.1 µm; image field, 1024 × 1024; FITC sola intensity, 25%; FITC exposure, 90 ms; TRITC sola intensity, 20%; TRITC exposure, 100 ms.

### Three-dimensional digital image reconstruction (fluorescence)

Widefield images were imported into Huygens Essential for deconvolution. Default parameters were used, with the exception of the deconvolution algorithm, which was set to QMLE. Deconvolved images were saved as 16-bit TIF files.

Deconvolved z-stacks were imported into Neurolucida 360 for semi-automated three-dimensional dendrite reconstruction. Next, the experimenter identified a 20- to 40-µm stretch of dendrite with clear dendritic spines and little to no overlap of other processes through the region of interest. The user-guided semiautomated directional kernel algorithm was used to trace the dendritic segment of interest. The trace generates a three-dimensional reconstruction of the dendrite, with points along the tree that can be moved in *x*, *y*, and *z* to ensure the reconstruction is as accurate as possible. Additionally, the thickness of the reconstruction at each point was modified as needed to fit the dendritic segment.

To reconstruct dendritic spines, the following parameters were used for the spine detector: outer range, 10 µm; minimum height, 0.5 µm; detector sensitivity, 100%; minimum count, eight voxels. The options to keep detected spines and filter image noise were selected. First, the detector was used to detect all spines. Then the experimenter modified each spine reconstruction as needed by increasing or decreasing the detector sensitivity to make the reconstruction larger or smaller, or using merge and slice tools to combine multiple points into one dendritic spine or split one reconstruction into multiple pieces. Incorrectly identified spines were deleted and any missed spines were added by increasing detector sensitivity.

Spines were classified as thin, stubby, mushroom, or filopodia using the predefined settings in Neurolucida 360. The criteria are as follows: head-to-neck ratio, 1.1; length-to-head ratio, 2.5; mushroom head size, 0.35 µm; filopodium length, 3.0 µm. Spines with a head-to-neck ratio > 1.1 and head diameter >0.35 µm were classified as mushroom. Spines were classified as filopodia or thin if head-to-neck ratio was <1.1 and either (1) length-to-head ratio was >2.5 or (2) head size was <0.35 µm. Of these, if the total length was >3.0 µm, the spine was classified as filopodia, and if <3.0 µm, as thin.

Reconstructions were then imported into Neurolucida Explorer for batch analysis. Dendritic spine measurements such as number of spines, length of each spine, etc. were exported to Microsoft Excel. Dendritic spine density, presented as number of spines per 10 µm, was calculated by dividing the number of dendritic spines by the length of the dendritic segment, then multiplying by 10.

### Figures

**Figure 1.**
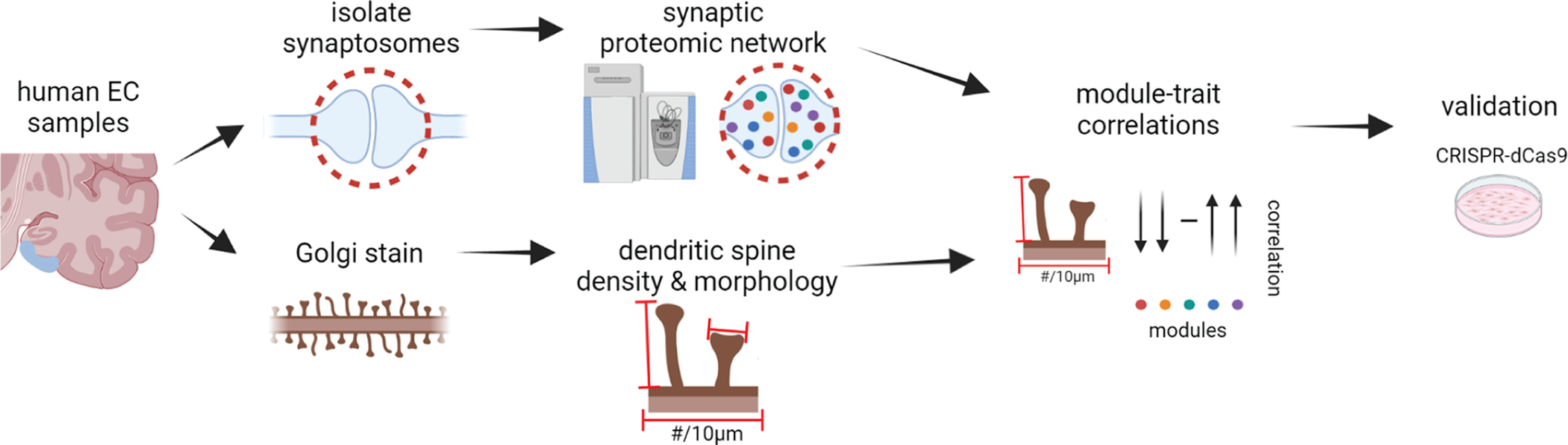
Overview of workflow. Synaptosomes were isolated from postmortem human BA28 entorhinal cortex (EC) and subjected to liquid chromatography tandem mass spectrometry-based proteomics. Weighted Gene Co-Expression Network Analysis (WGCNA) was used to generate a network of protein co-expression modules. BA28 EC samples were also Golgi stained and z-stacks of dendritic segments were imaged and digitally reconstructed to obtain measurements of dendritic spine density and morphology. Module eigenprotein values were correlated with dendritic spine metrics. The hub protein of a module significantly correlated with a dendritic spine metric would be selected for functional validation by CRISPR activation in rat primary hippocampal neurons.

Graphs were generated in GraphPad Prism (version 9.3.1) or R (version 4.1.1). The pipeline workflow (Fig. 1), CRISPRa schematic, and gRNA schematic were created with BioRender. Figures were assembled and generated in Adobe Illustrator (version 26).

### Experimental design and statistical analysis

#### MaxQuant for label-free proteome quantification

RAW data for all samples were analyzed using MaxQuant v1.6.0.1 with Thermo Foundation 2.0 for RAW file reading capability. The search engine Andromeda, integrated into MaxQuant, was used to build and search a concatenated target-decoy IPI/UniProt human reference protein database supplemented with APOE variants and β-amyloid (1–40 and 1–42) peptides sequences (retrieved April 20, 2015; 90 309 target sequences), plus 245 contaminant proteins from the common repository of adventitious proteins (cRAP) built into MaxQuant. Methionine oxidation (+15.9949 Da), and protein N-terminal acetylation (+42.0106 Da) were variable modifications (up to five allowed per peptide); cysteine was assigned a fixed carbamidomethyl modification (+57.0215 Da). Only fully tryptic peptides were considered with up to 2 miscleavages in the database search. A precursor mass tolerance of ± 20 ppm was applied before mass accuracy calibration and ±4.5 ppm after internal MaxQuant calibration. Other search settings included a maximum peptide mass of 6000 Da, a minimum peptide length of six residues, and 0.05 Da tolerance for high resolution MS/MS scans. Co-fragmented peptide search was enabled to deconvolute multiplex spectra. The false discovery rate (FDR) for peptide spectral matches, proteins, and site decoy fraction were all set to 1%. Quantification settings were as follows: re-quantify with a second peak finding attempt after protein identification has completed; match MS1 peaks between runs; a 0.7-min retention time match window was used after an alignment function was found with a 20 min RT search space. The quantitation method only considered razor plus unique peptides for protein level quantitation. Quantitation of proteins was performed using LFQ intensities given by MaxQuant.

##### Adjustment of batch and other co-variates

Batch adjustments were conducted using a median polish approach, as described (Johnson et al., 2020). Only proteins with <50% missing values were included in the final protein abundance matrix and no imputation of missing values was performed. One outlier control was omitted because of low signal abundance resulting in a higher frequency of missing values. Nonparametric bootstrap regression was performed on sex and PMI of the individual samples before differential and co-expression network approaches.

#### Experimental design and statistical analysis

Statistical analyses were performed in either GraphPad Prism (version 9.3.1) or R (version 4.1.1). In GraphPad Prism, outliers were detected using Grubbs' outlier test (α = 0.05). Data points from a normally distributed set that were deemed an outlier were excluded. Normality was determined using the Shapiro–Wilk normality test (α = 0.05). Homogeneity of variance was also assessed using Levene's test in R, or *F* test, Brown–Forsythe test, or Bartlett's test in GraphPad Prism. If both assumptions were met, parametric tests were used (unpaired *t* test, one-way ANOVA). If either assumption was not met, nonparametric tests were used (Mann–Whitney *U* test, Kruskal–Wallis test). Specifics of each statistical test are provided in the figure legends, along with information on sample sizes and error bars.

## Results

To determine whether integrating dendritic spine metrics with a human proteomic network could guide the identification of a protein involved in regulating dendritic spines, we first isolated synaptosome fractions from postmortem human Brodmann area (BA) 28 entorhinal cortex (EC) samples (Fig. 1). The cases used ranged from 40 to 96 years of age and exhibited varying degrees of AD pathology and cognitive function (Tables 1, 2). Individuals who were cognitively normal in life despite significant accumulation of AD pathology were referred to as cognitively normal individuals with AD pathology (CAD). Liquid chromatography coupled with tandem mass spectrometry (LC-MS/MS)-based proteomics was performed on the synaptosome fractions to generate a list of proteins present in the samples. Weighted Gene Co-expression Network Analysis (WGCNA) was used to generate a protein co-expression network. Dendritic spine density and morphology were measured on pyramidal neurons in layers 2 and three of BA28 EC from the same individuals. Protein co-expression module eigenprotein values were correlated with dendritic spine density and morphology to identify modules of proteins that may be involved in dendritic spine regulation. We selected one hub protein for functional validation in rat primary hippocampal neurons using neuron-optimized CRISPR activation (CRISPRa).

### Isolation and validation of human EC synaptosomes

**Figure 2.**
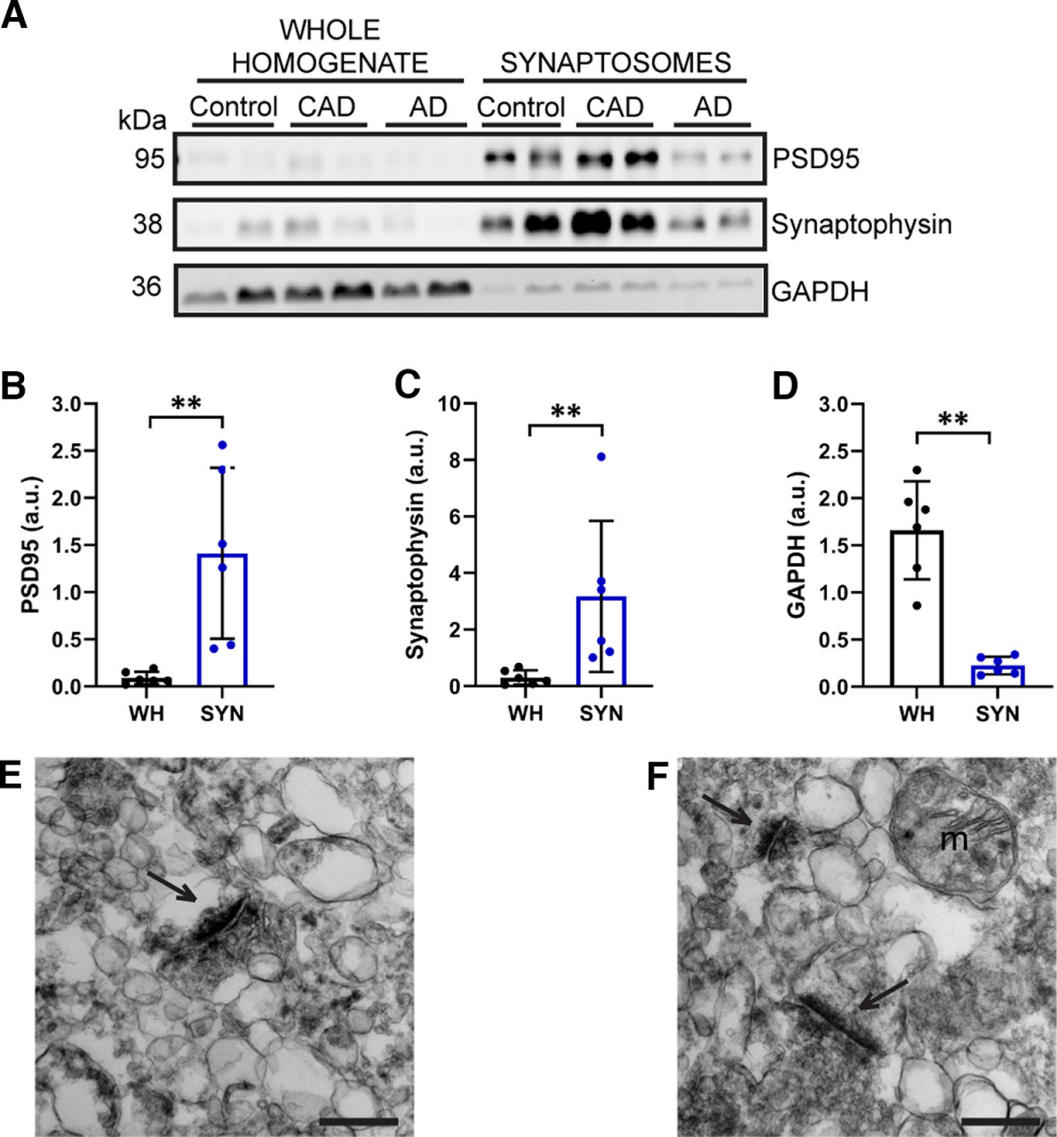
Isolation of synaptosomes from postmortem human entorhinal cortex. ***A***, Synaptosome fractions are enriched for PSD95 (postsynaptic marker) and synaptophysin (presynaptic marker), but have very low levels of GAPDH (nonsynaptic protein). *N* = 6 cases (2 control, 2 CAD, and 2 AD). ***B***, Quantification of PSD95 band in ***A*** showing that PSD95 is significantly enriched in synaptosome fractions compared with whole homogenates. Mann–Whitney *U* test (*U* = 0.0, *p* = 0.0022). ***C***, Quantification of synaptophysin band in ***A*** showing that synaptophysin is significantly enriched in synaptosome fractions compared with whole homogenates. Mann–Whitney *U* test (*U* = 0.0, *p* = 0.0022). ***D***, Quantification of GAPDH band in ***A*** showing that GAPDH is present at significantly lower levels in synaptosome fractions, as compared with whole homogenates. Mann–Whitney *U* test (*U* = 0.0, *p* = 0.0022). For ***B–D***, WH = whole homogenate, SYN = synaptosomes, a.u. = arbitrary units. Each point represents one case. Error bars represent the SD. ***E***, ***F***, Transmission electron micrographs (15000×) of the synaptosome fractions showing that these fractions contain synapses, as well as other cellular components, including mitochondria. Arrows indicate synapses, m = mitochondria. Scale bars = 400 nm.

Synaptosome fractions were biochemically isolated from 19 control, 7 CAD, and 31 AD postmortem EC samples (Tables 1, 2). These fractions were significantly enriched for the presynaptic and postsynaptic markers synaptophysin and PSD95, respectively, and contained very low amounts of the nonsynaptic protein, GAPDH (Fig. 2*A–D*). To further confirm the presence of synapses, transmission electron microscopy was performed on the synaptosome fractions. As expected, synapses were present, as well as myelin and mitochondria (Fig. 2*E*,*F*; Chin et al., 1989; Tai et al., 2012; Hesse et al., 2019).

In aging and AD, the EC is one of the first regions to develop tau NFTs (Hyman et al., 1984; Braak and Braak, 1991; Schöll et al., 2016; Maass et al., 2018). Tau phosphorylation sites that are associated with paired helical filaments, specifically T212/S214 and S422, are detected in EC synaptosomes from AD samples (Fein et al., 2008). Synaptosomes from other brain regions also contain higher levels of tau phosphorylated at S202, S396/S404, and S422 in AD compared with controls (Tai et al., 2012; Perez-Nievas et al., 2013; Bilousova et al., 2016). In the process of isolating synaptosomes, a biochemical fraction insoluble in aqueous buffer (henceforth referred to as the insoluble fraction) was also generated (Hallett et al., 2008). In frontal and temporal cortices, AD brains contain more total and phosphorylated aqueous buffer-insoluble and detergent-insoluble tau compared with control brains (Hanger et al., 1991; Koss et al., 2018). Thus, we expected synaptosome and insoluble fractions from our EC samples to contain greater amounts of phospho-tau when isolated from AD cases compared with controls. To validate these expectations, we quantified phospho-sites present early in disease progression (S199 and T231) and in paired helical filaments (S396; Bramblett et al., 1993; Maurage et al., 2003; Luna-Muñoz et al., 2007) using ELISAs for human tau phosphorylated at each site.

**Figure 3.**
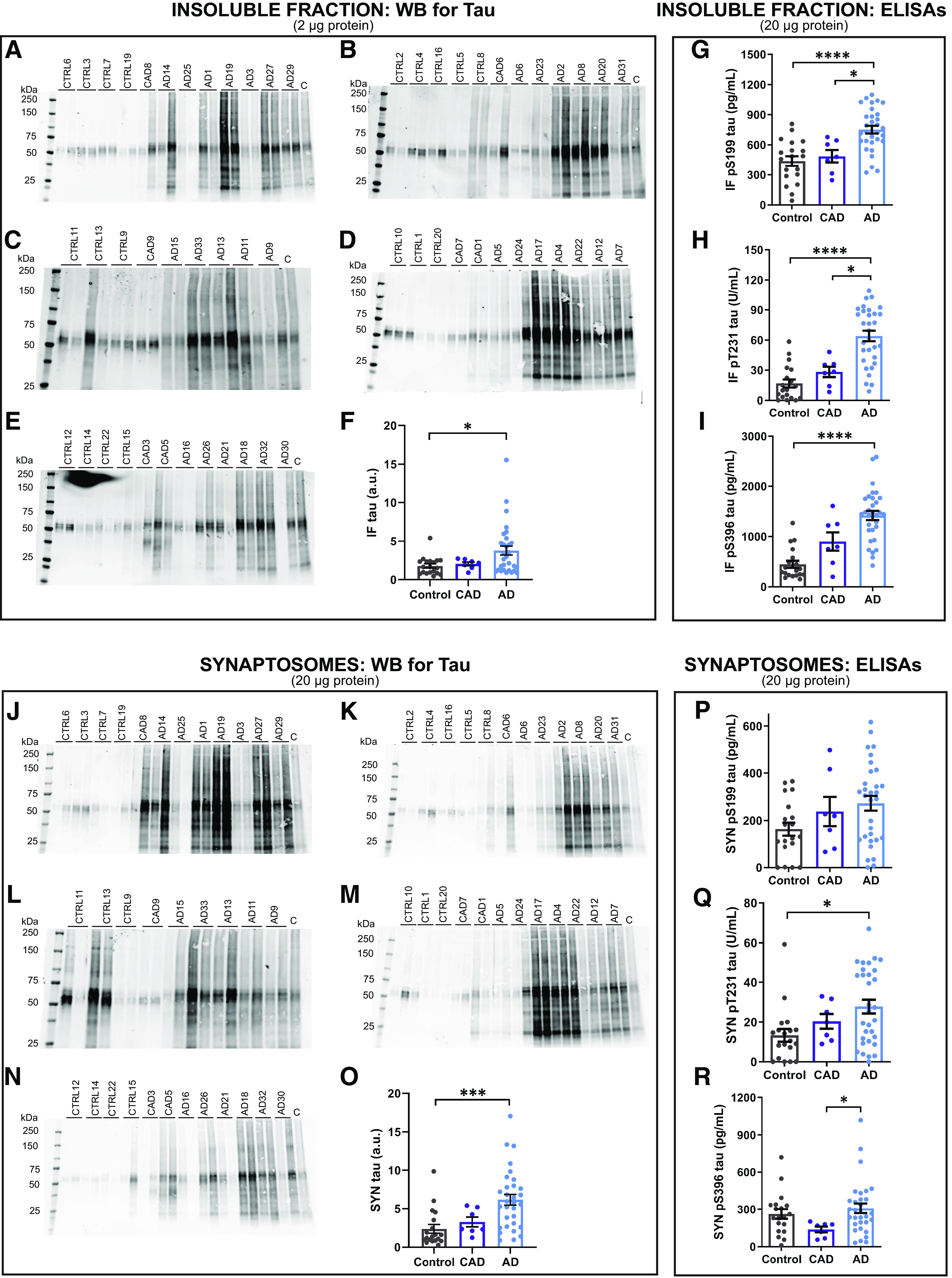
Tau accumulates in synaptosomes in AD entorhinal cortex samples. ***A–E***, Representative Western blottings for tau in human EC insoluble fractions. ***C*** indicates a control sample loaded on each Western blotting. ***F***, Quantification of Western blottings of insoluble fractions probed for tau. AD cases harbor significantly greater amounts of tau in the insoluble fraction in the EC compared with controls. Kruskal–Wallis test (H(2) = 7.278, *p* = 0.0263) with Dunn's multiple comparisons test. *p = 0.021. ***G***, AD insoluble fractions contained significantly more tau phosphorylated at S199 compared with insoluble fractions from both control and CAD cases. One-way ANOVA (*F*_(2,54)_ = 14.68, *p* = 8.1 × 10^−6^) with Tukey's multiple comparisons test. *p = 0.011, ****p = 1.2 × 10^−5^. ***H***, Insoluble tau phosphorylated at T231 was significantly increased in AD cases compared with control and CAD cases. Kruskal–Wallis test (H(2) = 27.62, *p* = 1.0 × 10^−6^) followed by Dunn's multiple comparisons test. *p = 0.037, ****p = 9.2 × 10^−7^. ***I***, AD insoluble fractions contained significantly more tau phosphorylated at S396 compared with controls. Kruskal–Wallis test (H(2) = 29.49, *p* = 4.0 × 10^−7^) with Dunn's multiple comparisons test. ****p = 1.9 × 10^−7^. ***J–N***, Representative western blot of tau in human EC synaptosomes. C indicates a control sample loaded on each Western blotting. ***O***, Quantification of Western blottings of synaptosome fractions probed for tau. AD cases harbor significantly more tau in EC synaptosomes compared with controls. Kruskal–Wallis test (H(2) = 17.23, *p* = 0.0002) with Dunn's multiple comparisons test. ***p = 0.0001. ***P***, Synaptosomal tau phosphorylated at S199 is not significantly different among control, CAD, and AD cases. ***Q***, AD synaptosomes contain significantly more synaptosomal tau phosphorylated at T231 compared with controls. Kruskal–Wallis test (H(2) = 7.436, *p* = 0.0243) with Dunn's multiple comparisons test. *p = 0.02. ***R***, AD synaptosomes contain significantly more synaptosomal tau phosphorylated at S396 compared with CAD cases. Kruskal–Wallis test (H(2) = 6.782, *p* = 0.0337) with Dunn's multiple comparisons test. *p < 0.028. *N* = 57 (19 control, 7 CAD, and 31 AD cases). Each point represents one case. Error bars represent SEM.

Consistent with prior observations, we observed that AD cases harbored significantly more tau in the EC insoluble fraction than controls, as assessed by Western blot densitometry analysis (Fig. 3*A–F*). High molecular weight tau oligomers and low molecular weight fragments were observed in insoluble fractions from AD samples, but not controls. The Western blotting tau banding pattern of insoluble fractions from some CAD cases resembled that which was observed in controls, whereas other CAD cases exhibited the high-molecular and low-molecular weight tau smears that were abundant in AD patients. To quantify phospho-tau in the insoluble fractions, we used ELISAs specific for tau phosphorylated at S199, T231, or S396. Levels of pS199, pT231, and pS396 tau from AD cases were increased significantly compared with controls. AD brains also contained significantly higher levels of pS199 and pT231 tau compared with CAD samples (Fig. 3*G–I*). Overall, insoluble fractions from AD cases contained greater amounts of phosphorylated tau compared with insoluble fractions from controls, indicating that our biochemical approaches yielded results comparable to past findings (Hanger et al., 1991; Koss et al., 2018).

Next, we measured tau in EC synaptosomes. First, we performed densitometry analysis for total tau on Western blottings. Synaptosomes from AD cases contained significantly more tau compared with controls (Fig. 3*J–O*). More high molecular weight tau oligomers and low molecular weight fragments were observed in AD synaptosomes compared with controls. Next, we quantified synaptosomal tau phosphorylated at S199, T231, or S396 by ELISA. Among control, CAD, and AD cases, there was not a significant difference in levels of pS199 tau, a phospho-site that occurs early in disease progression (Fig. 3*P*). However, AD synaptosomes contained significantly higher levels of pT231 tau, another early disease state phospho-site, compared with controls (Fig. 3*Q*). Levels of pS396 tau, associated with paired helical filaments, were significantly higher in synaptosomes from AD cases compared with CAD (Fig. 3*R*).

**Figure 4.**
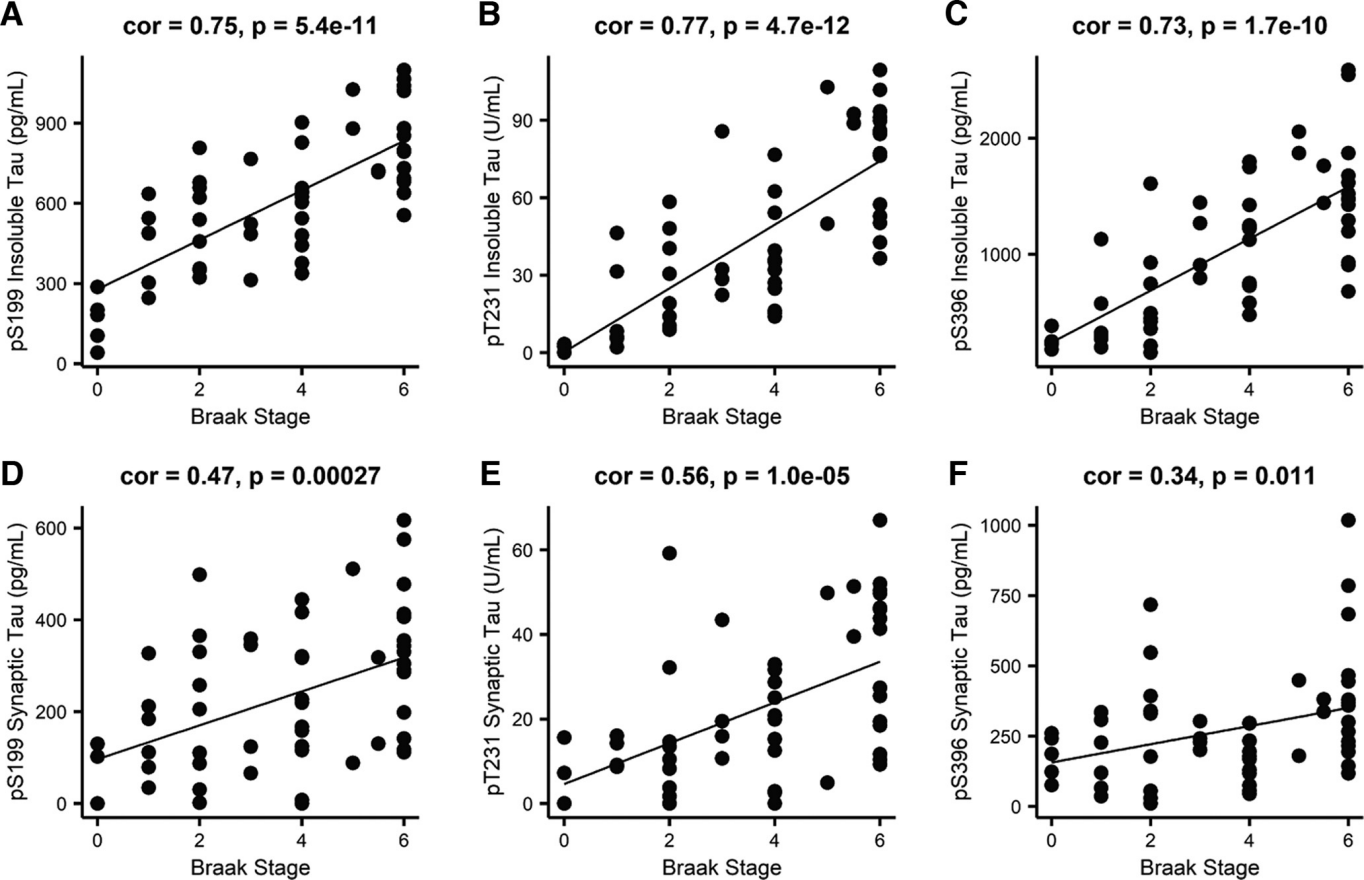
Biochemical measurements of phospho-tau strongly correlate with Braak stage. Pearson correlations between Braak stage and insoluble tau phosphorylated at (***A***) S199, (***B***) T231, and (***C***) S396, and synaptosomal tau phosphorylated at (***D***) S199, (***E***) T231, and (***F***) S396. *N* = 56 (19 control, 7 CAD, and 30 AD cases). Each point represents one case.

Finally, we determined whether the phospho-tau measurements correlated with Braak stage. Across all cases, insoluble phospho-tau levels and synaptosome phospho-tau levels correlated significantly with Braak stage (Fig. 4). Collectively, these findings indicate that our biochemical fractionation of samples and case-specific disease traits accurately reflect expectations of tau-related increases that are highly characteristic of AD.

### Generation of human entorhinal cortex synaptic protein co-expression network

**Figure 5.**
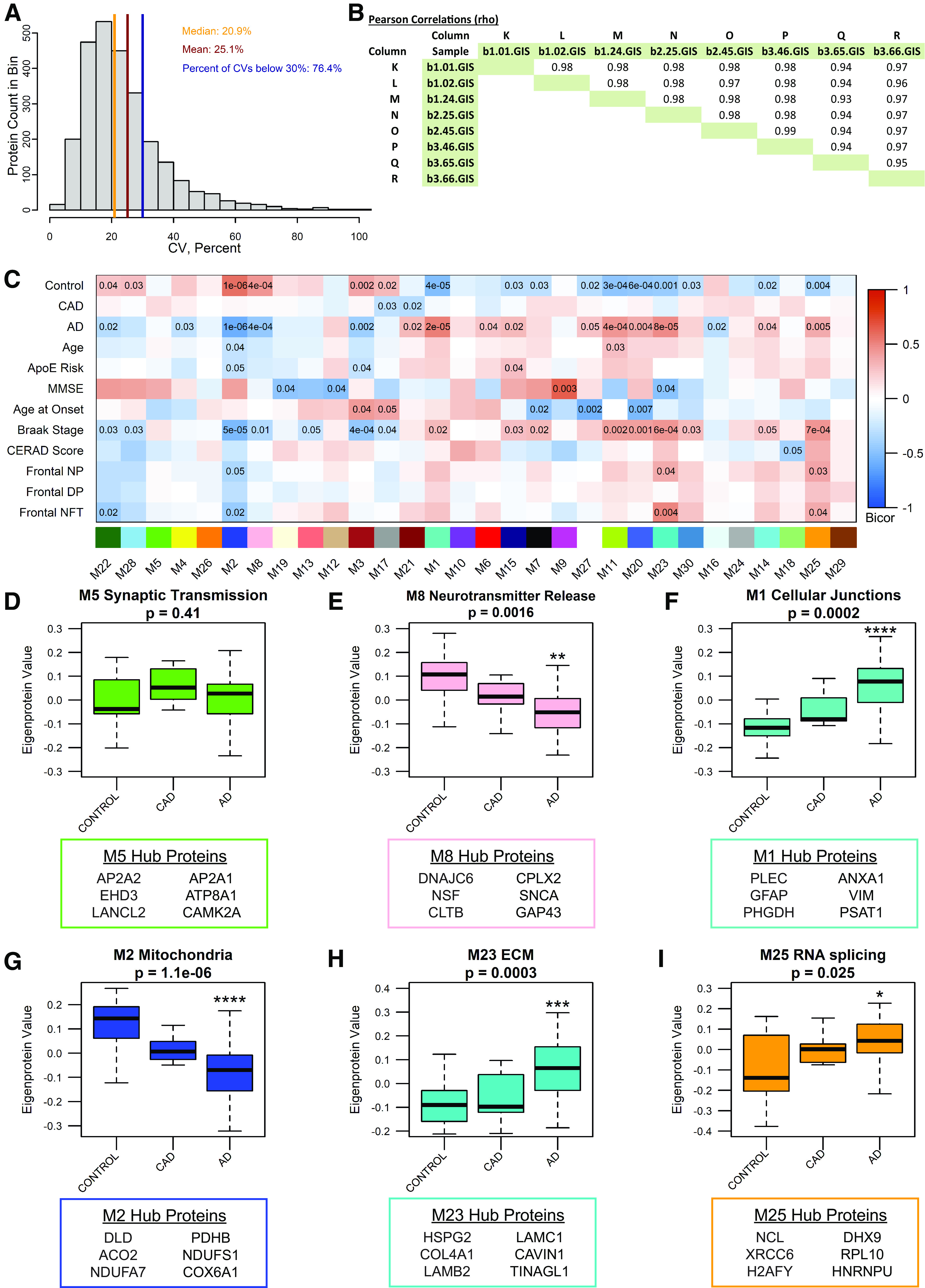
The entorhinal cortex synaptosomal proteome is altered in AD. ***A***, The coefficient of variance (CV) for proteins having at least 50% nonmissing values in measurements of the global internal standards (GIS). The GIS are a pooled peptide mixture reference sample that serves as technical replicates. ***B***, The Pearson correlation ρ values from least squares fit line calculations for all pairwise nonmissing proteins measured in GIS replicates. ***C***, Biweight midcorrelation (bicor) for each module's eigenprotein abundance with case demographic and pathology data. Uncorrected p-values are shown for correlations with *p* < 0.05. ***D***, Module 5, enriched for synaptic transmission-related gene ontology (GO) terms, is not differentially expressed between groups. ***E***, Module 8 is described by GO terms related to synaptic transmission and neurotransmitter release. Module 8 is differentially expressed among groups, with reduced eigenprotein abundance in AD compared with control. One-way ANOVA (*F*_(2,54)_ = 7.27, *p* = 0.0016) with Tukey's multiple comparisons test. **p = 0.0011. ***F***, Module 1, containing cellular junction-related proteins, is differentially expressed among groups, with significantly higher expression in AD compared with control. Kruskal–Wallis test (H(2) = 17.60, *p* = 0.0002), followed by pairwise Wilcoxon rank sum exact test with Benjamini-Hochberg adjustment. ****p = 6.1 × 10^−5^. ***G***, The mitochondrial module, Module 2, eigenprotein expression differs among groups, with significantly reduced expression in AD compared with control. One-way ANOVA (*F*_(2,54)_ = 17.94, *p* = 1.06 × 10^−6^) with Tukey's multiple comparisons test. ****p = 6.0 × 10^−7^. ***H***, Module 23 is described by GO terms related to the extracellular matrix, and is differentially expressed among groups, with significantly higher expression in AD compared with control. One-way ANOVA (*F*_(2,54)_ = 9.413, *p* = 0.0003) with Tukey's multiple comparisons test. ***p = 0.00,036. ***I***, Module 25 is enriched for GO terms related to RNA splicing. Module 25 eigenprotein expression differs among groups, with significantly higher expression in AD compared with control. Kruskal–Wallis test (H(2) = 7. 353, *p* = 0.025), followed by pairwise Wilcoxon rank sum exact test with Benjamini-Hochberg adjustment. *p = 0.032. For ***D–I***, *N* = 57 (19 Control, 7 CAD, 31 AD). Error bars represent the 25th and 75th percentiles. * indicates significant difference compared with control group. The top 6 hub proteins for each module are listed below the corresponding boxplot. See Extended Data Tables 5-1 and 5-2 for lists of module members and connectivity values (kME), and gene ontologies for each module.

Next, synaptosomes were analyzed by MS-based proteomics using label-free quantitation (LFQ). The MS data were processed using a common pipeline to arrive at 2260 total quantified proteins. Only proteins with fewer than 50% missing global pooled peptide reference standard sample measurements were included in our analyses. We assessed the coefficients of variation (CV) for 2623 proteins having at least four of eight nonmissing values (50% measured) among the global standards, which were among the 66 total injections making up the RAW LC-MS/MS LFQ files. The CVs were plotted as a histogram for the range 0–100% CV, and the percent of the area under the curve was calculated below thresholds of the median CV, mean CV, and for CV <30%. 50% of proteins had a CV <20.9%, the mean CV was 25.1%, and 76.4% of the 2623 well-measured proteins were below a 30% CV threshold. Based on this, we concluded that CVs representing underlying variation of the technical replicate data were predominantly below 25% and that the vast majority was below 30% (Fig. 5*A*). Next, the population of log_2_-transformed LFQ intensities for all pairs of single protein measurements in each pair of single injection-specific LC-MS/MS sample quantifications were plotted. Log_2_-transformed LFQ intensity in the first sample of each pair was plotted on the *x*-axis. The corresponding measurement in the second sample of each pair was plotted on the *y*-axis of the pair-specific scatterplots, indicating the Pearson correlation ρ value (Fig. 5*B*). From this analysis, we conclude by these highly correlated sets of common protein measurements across pairs of samples, that our reproducibility and precision of measurement by the LFQ approach are excellent. Of the 2260 proteins identified and used to generate a protein co-expression network using Weighted Gene Co-Expression Network Analysis (WGCNA; Langfelder and Horvath, 2008); 1942 proteins (86%) were co-expressed in modules. This algorithm examines the expression pattern of each protein across all samples and groups proteins with similar expression patterns together into protein co-expression modules. The resulting network consisted of 30 protein co-expression modules, or groups of proteins with similar expression patterns across cases (Fig. 5*C*). Each module was assigned a different color to aid in data visualization. Modules ranged in size from 15 proteins (Module 30) to 194 proteins (Module 1; Extended Data Table 5-1). WGCNA also assesses connectivity of proteins within modules by determining how strongly the expression pattern of each protein correlates with the expression pattern of every other protein in the module. These correlations are then ranked by strength of correlation, with the most highly correlated proteins being considered the “hub” proteins and thought of as being drivers of the module as a whole.

10.1523/JNEUROSCI.2102-22.2023.tab5-1 Extended Data Table 5-1 Module members are shown for all modules, ranked from highest to lowest kME (connectivity) within each module. Download Table 5-1, XLSX file.

To ensure that the input peptides generated from synaptosome fractions and the output WGCNA analysis represented synapse biology, we explored the network to identify synaptic modules. Four modules (Modules 4, 5, 7, and 8) contained proteins with known synaptic localization or function, based on the gene ontologies (GOs). GO enrichment analysis using GO-Elite was used to identify biological processes, molecular functions, and cellular components enriched in each module. Based on GO terms, Module 5 is involved in synaptic transmission (Extended Data Table 5-2), but is not differentially expressed among control, CAD, and AD cases. Hub proteins for Module 5 include, but are not limited to, AP2A2, EHD3, LANCL2, AP2A1, ATP8A1, and CAMK2A (Fig. 5*D*; Extended Data Table 5-1). These proteins are involved in various processes relevant to synaptic function. AP2A2 and AP2A1 encode the α subunit of the adaptor protein complex 2, which is a critical component of clathrin-mediated endocytosis (Nelson et al., 2020). ATP8A1 is involved in plasma membrane phospholipid distribution, and alteration of ATP8A1 expression affects synaptic activity (Kerr et al., 2016). CAMK2A is a mediator of long-term potentiation (Barria and Malinow, 2005). Other notable synaptic proteins present in Module 5 include BSN (Sanmartí-Vila et al., 2000; Winter et al., 1999), SYNGAP1 (Llamosas et al., 2020), GRIA2 (GluA2 AMPA receptor subunit), and GRIN1 (GluN1 NMDA receptor subunit), among others. Another module containing proteins involved in synaptic transmission and neurotransmitter release is Module 8 (Extended Data Table 5-2), which is significantly reduced in AD compared with controls (Fig. 5*E*). The Module 8 hub proteins include DNAJC6, NSF, CLTB, CPLX2, SNCA, and GAP43 (Fig. 5*E*; Extended Data Table 5-1). DNAJC6 and CLTB are involved in clathrin-mediated endocytosis (Nguyen et al., 2019; Redlingshöfer et al., 2020); NSF, CPLX2, and SNCA play important roles in synaptic vesicle exocytosis (Südhof and Rothman, 2009; Südhof and Rizo, 2011); and GAP43 is involved in long-term depression and AMPA receptor endocytosis (Han et al., 2013).

10.1523/JNEUROSCI.2102-22.2023.tab5-2 Extended Data Table 5-2 Gene ontology output (limited to biological processes, molecular functions, and cellular components) from GO-Elite is shown for all modules. Download Table 5-2, XLSX file.

We further interrogated our synaptosome proteomic network by assessing whether the analysis exhibited expected correlations with AD-relevant clinical and pathologic traits. Module eigenprotein expression (first principal component of module protein expression level) was correlated with case clinical and pathology traits (Fig. 5*C*). Twelve modules were opposing in expression values among control and AD cases, indicating a notable alteration in the proteome of EC synaptosomes with AD diagnosis. Module expression patterns largely matched for AD and Braak stage, which is expected since (1) AD progression correlates strongly with Braak stage and (2) tau pathology initiates in the EC (Duyckaerts et al., 1997; Nelson et al., 2012). Next, we identified modules that may be important for AD based on module correlations with AD pathology scores. Modules 2, 23, and 25 were significantly correlated with measures of Aβ and tau pathology, and Module 1 was strongly correlated with AD (Fig. 5*C*). Eigenprotein abundance of Modules 1, 23, and 25 was significantly higher in AD compared with controls, while eigenprotein abundance of Module 2 was significantly lower in AD than controls (Fig. 5*F–I*). Module 1, with hub proteins including PLEC, GFAP, PHGDH, ANXA1, VIM, and PSAT1, is relevant to cellular junctions (Fig. 5*F*; Extended Data Tables 5-1, Tables 5-2). Module 2 is a mitochondrial module, containing hub proteins such as DLD, ACO2, NDUFA7, PDHB, NDUFS1, and COX6A1, which play a role in the electron transport chain and other cellular bioenergetic processes (Fig. 5*G*; Extended Data Tables 5-1, Tables 5-2). Module 23 contains structural, extracellular matrix proteins, including HSPG2, COL4A1, LAMB2, LAMC1, CAVIN1, and TINAGL1 (Fig. 5*H*; Extended Data Tables 5-1, Tables 5-2). Module 25 is composed of proteins important for RNA binding/splicing and gene expression, including NCL, XRCC6, H2AFY, DHX9, RPL10, and HNRNPU (Fig. 5*I*; Extended Data Tables 5-1, Tables 5-2). Notably, modules of proteins with similar functions based on GO terms have been associated with AD in other proteomic co-expression networks (Seyfried et al., 2017; Zhang et al., 2018; Johnson et al., 2020). Hence, these findings indicate that our proteomic network has captured synaptic biology as well as neurobiology relevant to AD.

### Dendritic spine density and morphology in human entorhinal cortex

**Figure 6.**
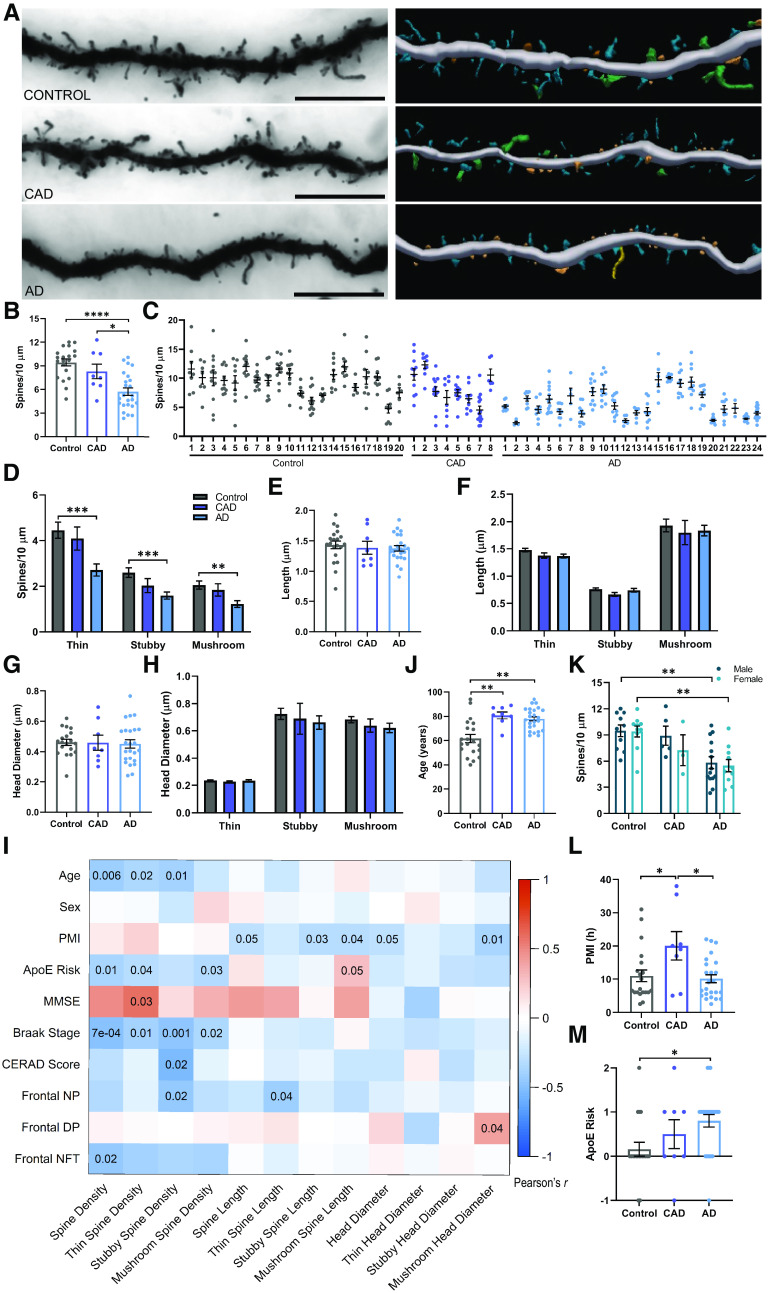
Entorhinal cortex spine density is reduced in AD. ***A***, Representative brightfield images (left) and three-dimensional reconstructions (right) of Golgi-stained dendrites from control (top), CAD (middle), and AD (bottom) cases. Scale bar = 10 µm. In the reconstructions, blue = thin, orange = stubby, green = mushroom, and yellow = filopodia. ***B***, Dendritic spine density is reduced in AD, but maintained in CAD cases. One-way ANOVA (*F*_(2,49)_ = 15.36, *p* < 7.0 × 10^−6^) followed by Tukey's multiple comparisons test. *p = 0.019, ****p = 5.0 × 10^−6^. ***C***, Dendritic spine density per dendrite for each case. Case numbering corresponds to Tables 1 and 2. Each point represents one dendrite. *N* = 3–17 dendrites per case. ***D***, Thin, stubby, and mushroom spine densities are reduced in AD compared with controls. One-way ANOVA (Thin: *F*_(2,49)_ = 8.422, *p* = 0.0007; Stubby: *F*_(2,49)_ = 7.680, *p* = 0.0013; Mushroom: *F*_(2,49)_ = 7.115, *p* = 0.0019) with Tukey's multiple comparisons test. Thin ***p = 0.0007, Stubby ***p = 0.0008, Mushroom **p = 0.0017. ***E***, No differences in dendritic spine length were observed between groups. ***F***, Dendritic spine length of thin, stubby, and mushroom spines did not differ between groups. ***G***, No differences in dendritic spine head diameter were observed between groups. ***H***, Head diameter on thin, stubby, and mushroom spines was similar between groups. ***I***, Pearson correlations between dendritic spine measurements and case demographic and pathology data. Uncorrected p-values are shown for correlations with *p* < 0.05. PMI = postmortem interval, MMSE = Mini-Mental State Examination, NP = neuritic plaques, DP = diffuse plaques, NFT = neurofibrillary tangles. ***J***, CAD and AD cases were significantly older than controls. Kruskal–Wallis test (H(2) = 16.18, *p* = 0.0003) with Dunn's multiple comparisons test. Control versus CAD **p = 0.004, Control versus AD **p = 0.0014. ***K***, There were no sex differences in dendritic spine density – males and females exhibited a similar reduction in dendritic spine density in the EC in AD. Two-way ANOVA (main effect of diagnosis: *F*_(2,46)_ = 14.56, *p* = 1.3 × 10^−5^) with Šídák's multiple comparisons test. Male **p = 0.0062, Female **p = 0.0071. ***L***, The PMI before brain collection was longer for CAD cases compared with controls and AD cases. One-way ANOVA (*F*_(2,49)_ = 4.913, *p* = 0.0114) with Tukey's multiple comparisons test. CAD versus Control *p = 0.024, versus AD *p = 0.01. ***M***, ApoE risk is higher in AD cases, compared with controls. Calculation of ApoE risk is described in Materials and Methods. One-way ANOVA (*F*_(2,49)_ = 4.114, *p* = 0.0223) with Tukey's multiple comparisons test. *p = 0.017. *N* = 20 Control, 8 CAD, and 24 AD cases. *N* = 52 (20 control, 8 CAD, and 24 AD), unless specified otherwise. Each point represents one case. Error bars indicate SEM.

Postmortem human BA28 EC samples from 20 control, 8 CAD, and 24 symptomatic AD cases were selected based on availability of BA28 tissue, as well as adherence to criteria delineating the three diagnostic groups. These criteria include MMSE cognitive test scores, CERAD scores for Aβ plaque pathology, and Braak staging of tau NFT pathology. Further details are provided in the Methods section. Paraformaldehyde-fixed tissue sections were Golgi-stained, imaged with brightfield microscopy, and digitally reconstructed using Neurolucida 360 (Fig. 6*A*).

AD cases exhibited a significant reduction in dendritic spine density in the EC, compared with controls and CAD cases (Fig. 6*B*). Spine density per dendrite for each individual is shown in Figure 6*C*. Controls and CAD cases exhibited comparable spine density in the EC, supporting the hypothesis that maintenance and preservation of dendritic spines is critical for retaining cognitive function in the presence of AD pathology (Boros et al., 2017). The primarily used morphologic spine subclasses are thin, stubby, and mushroom (Peters and Kaiserman-Abramof, 1969). These subclasses are based on ratios of spine head diameter and spine length measurements. Thin spines have short necks and small heads, with smaller synapses. Conversely, mushroom spines have longer necks and larger heads, which house postsynaptic densities containing a greater number of neurotransmitter receptors (Matsuzaki et al., 2001). Stubby spines are transitional structures with a short neck and wide head. Here, we observed a loss of thin, stubby, and mushroom spines in AD cases compared with controls, indicating a global reduction of spines in EC, rather than loss of a specific spine type (Fig. 6*D*). Next, we examined dendritic spine length and head diameter. No differences in dendritic spine length were detected among control, CAD, and AD cases (Fig. 6*E*,*F*). Additionally, head diameter was comparable between groups (Fig. 6*G*,*H*).

Next, dendritic spine density and morphology measurements were correlated with case demographic and pathology data. We observed a significant inverse correlation of spine density with age, which was also observed for thin and stubby spine subclasses (Fig. 6*I*). While the control group was significantly younger than the CAD and AD groups (Fig. 6*J*), dendritic spine density was comparable between the control and CAD cases, suggesting that age alone is not entirely responsible for spine loss in the AD group. Additionally, density of thin spines exhibited a significant positive correlation with MMSE scores (Fig. 6*I*). This is highly consistent with findings in nonhuman primates showing a positive association between thin spine density and cognitive performance (Dumitriu et al., 2010). Sex was not associated with any dendritic spine measurements (Fig. 6*I*,*K*). The PMI was significantly greater in the CAD group compared with the control and AD groups (Fig. 6*L*). While PMI was significantly inversely correlated with spine length and head diameter (Fig. 6*I*), spine length and head diameter were not significantly different among groups (Fig. 6*E–H*). As expected, the ApoE risk was greater in the AD group compared with controls (Fig. 6*M*), and Braak stage displayed a significant inverse correlation with spine density (Fig. 6*I*), which was observed in previous studies of the DLPFC (Boros et al., 2017).

### Correlation of dendritic spine density and morphology with synaptic protein modules

**Figure 7.**
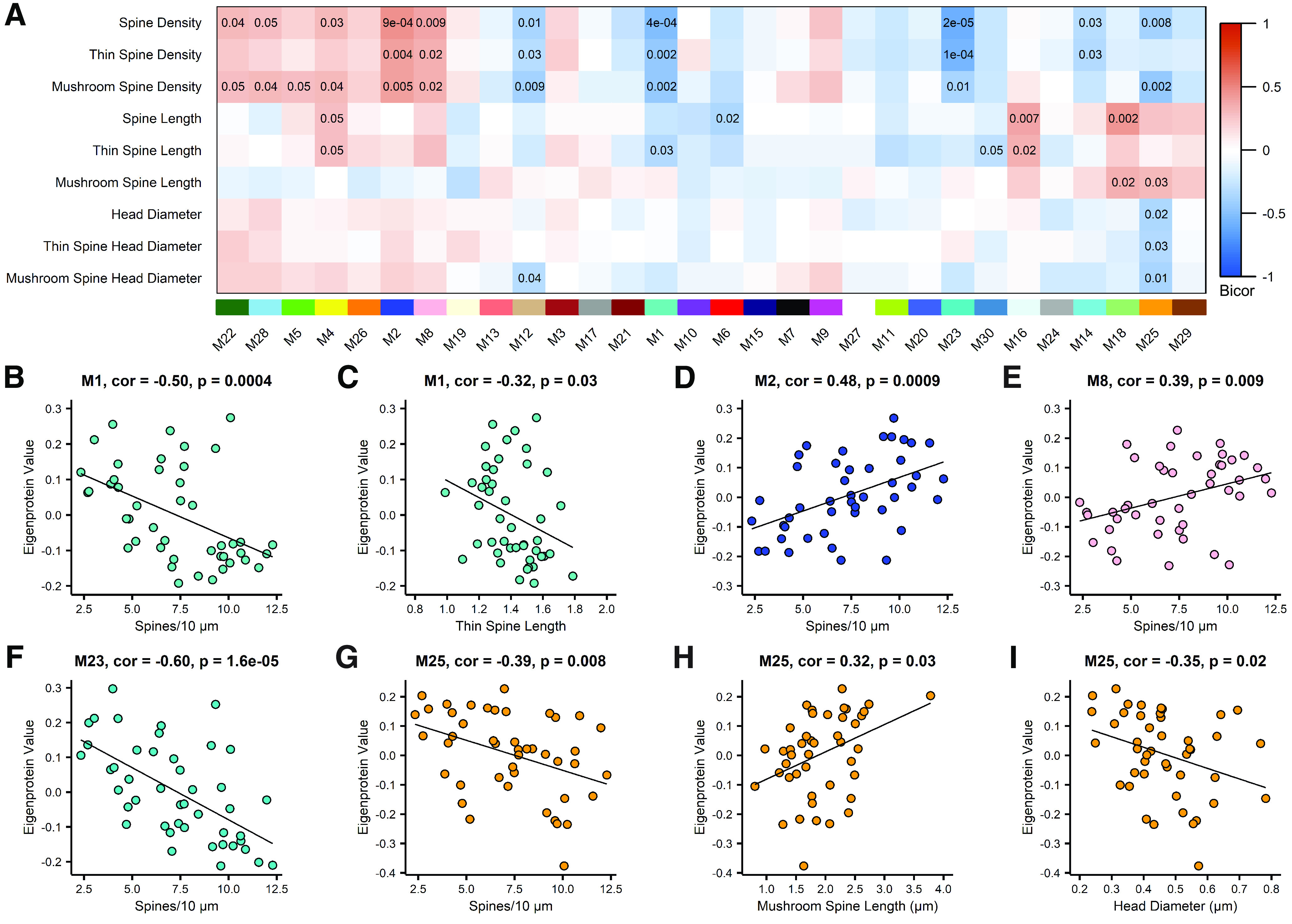
Protein co-expression modules correlate with dendritic spine density and morphology. ***A***, Biweight midcorrelations (bicor) between module eigenprotein expression and dendritic spine measurements. Uncorrected p-values are shown for correlations with *p* < 0.05. ***B***, There is a significant inverse correlation between Module 1 eigenprotein abundance and spine density. ***C***, Module 1 eigenprotein abundance is significantly inversely correlated with thin spine length. ***D***, Eigenprotein expression of Module 2 is significantly positively correlated with spine density. ***E***, Module 8 eigenprotein abundance exhibits a significant positive correlation with spine density. ***F***, Module 23 eigenprotein abundance exhibits a significant inverse correlation with spine density. ***G***, Module 25 is significantly inversely correlated with spine density. ***H***, Eigenprotein expression of Module 25 is positively correlated with mushroom spine length. ***I***, There is a significant inverse correlation between Module 25 eigenprotein expression and dendritic spine head diameter. Correlations were assessed by bicor. Each point represents one case. *N* = 45 (17 Control, 6 CAD, 22 AD).

Dendritic spine density and morphology measurements were integrated with the network to identify modules that may regulate specific aspects of dendritic spine biology. This was achieved by correlating dendritic spine measurements with module eigenprotein expression for each module (Fig. 7*A*). Several modules, including Modules 1, 2, 8, 23, and 25, were significantly correlated with multiple spine traits. Module 1 (cellular junctions) was inversely correlated with spine density, thin spine density, mushroom spine density, and thin spine length (Fig. 7*A–C*). Module 2 (mitochondria) exhibited significant positive correlations with overall spine density, as well as thin and mushroom spine density (Fig. 7*A*,*D*). Module 8 (neurotransmitter release) was positively correlated with overall spine density, as well as density of thin and mushroom spines (Fig. 7*A*,*E*). The extracellular matrix module (Module 23) was inversely correlated with overall spine density, thin spine density, and mushroom spine density (Fig. 7*A*,*F*). The RNA splicing module (Module 25) was inversely correlated with overall spine density and mushroom spine density; positively correlated with mushroom spine length; and inversely correlated with overall head diameter, as well as thin spine and mushroom spine head diameters (Fig. 7*A*,*G–I*). Notably, Module 8 (neurotransmitter release) displayed a positive correlation with overall spine density, providing evidence that our approach can identify relevant neurobiological associations between protein modules and integrated cellular traits.

### Protein target validation of TWF2 on dendritic spine morphology

**Figure 8.**
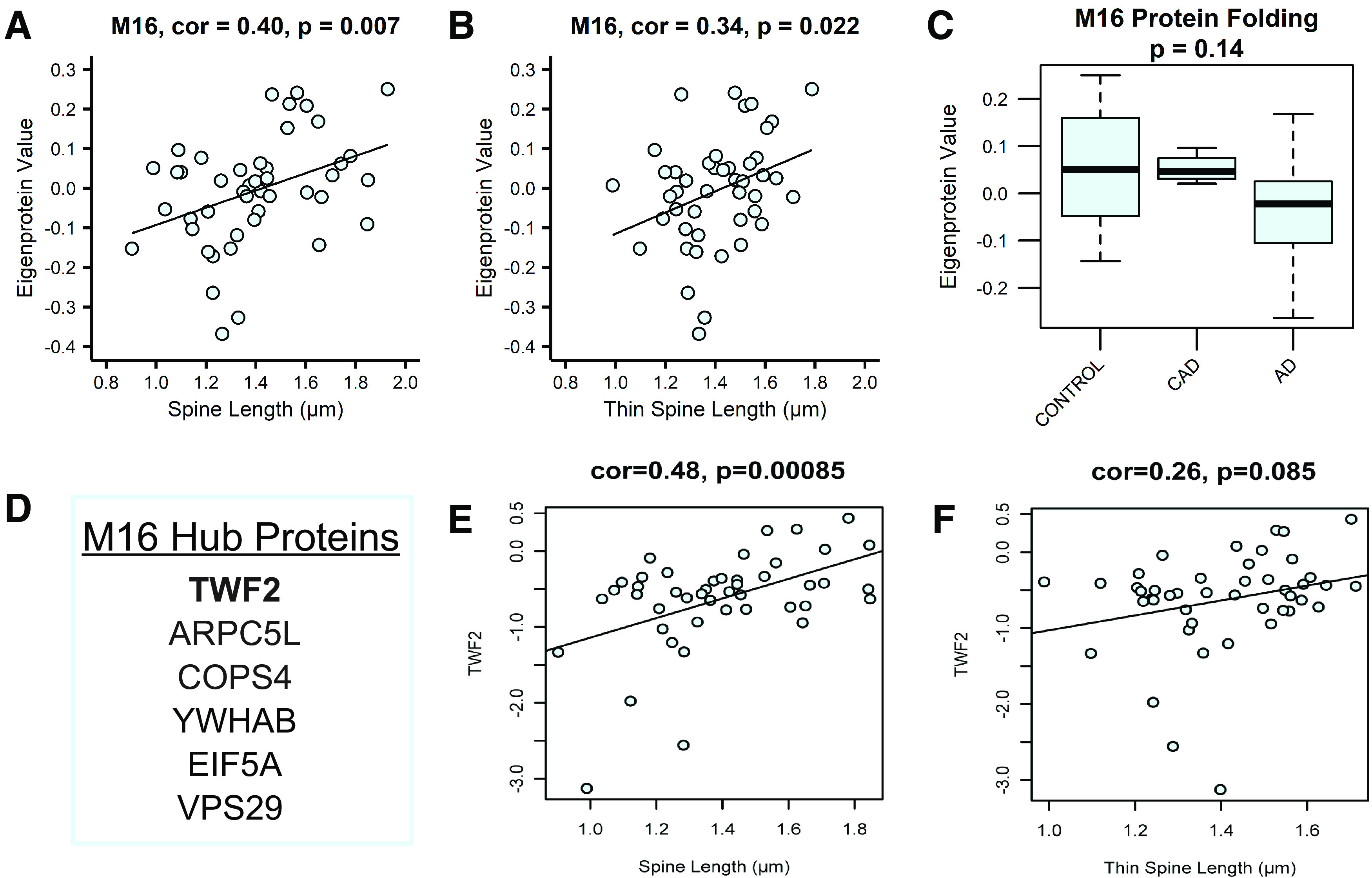
Relative protein abundance of TWF2 positively correlates with spine length. ***A***, Module 16 eigenprotein abundance exhibits a significant positive correlation with dendritic spine length. ***B***, Module 16 eigenprotein abundance is significantly positively correlated with thin spine length. Each point represents one case. *N* = 45 (17 Control, 6 CAD, 22 AD). ***C***, Gene ontology (GO) for Module 16 indicates involvement in protein folding. Module 16 eigenprotein abundance does not differ among control, CAD, and AD synaptosomes. *N* = 57 (19 Control, 7 CAD, 31 AD). Error bars indicate the 25th and 75th percentiles. ***D***, The top 6 hub proteins for Module 16 are shown. TWF2 is the top hub protein. ***E***, Log_2_-transformed relative protein abundance of TWF2 positively correlates with spine length. ***F***, Log_2_-transformed relative protein abundance of TWF2 was plotted against thin spine length. For ***E***, ***F***, points represent individual cases and the best fit line was determined via linear model. For ***A***, ***B***, ***E***, ***F***, correlations were assessed by biweight midcorrelation (bicor).

To test whether manipulating hub protein expression can modulate spine traits in an experimental model system, we selected Module 16, which was positively and exclusively correlated with overall dendritic spine length and thin spine length (Figs. 7*A*, 8*A*,*B*). The correlation between Module 16 and overall dendritic spine length was likely driven by thin spines, given that thin spines were the most abundant spine class on EC dendrites (Fig. 6*D*). Moreover, Module 16 was selected for target validation because Module 16 was not differentially expressed among control, CAD, and AD cases (Fig. 8*C*). We hypothesized that if the top hub protein abundance was presumably not influenced by disease aspects, then changes in abundance of the hub protein would more directly alter correlated cellular traits. Notably, the log_2_-transformed protein abundances of Actin β (ATCB) were similar across control, CAD, and AD cases.

GO analysis indicated that Module 16 harbored proteins involved in posttranslational protein folding and actin filament polymerization. The top hub protein of Module 16 was Twinfilin-2 (TWF2; Fig. 8*D*), an actin binding protein that influences cytoskeletal dynamics (Palmgren et al., 2001; Vartiainen et al., 2003; Yamada et al., 2007). TWF2 is present in mouse brain (Vartiainen et al., 2003) and regulates the growth of projections on rat primary cortical neurons (Yamada et al., 2007), but whether TWF2 regulates dendritic spine density or morphology, remains unknown. Next, the log_2_-transformed protein abundances of TWF2 across all individuals, regardless of case diagnosis, was plotted to test associations with spine length and thin spine length. Correlations were assessed by biweight midcorrelation, where each point represents one case. A significant positive correlation of TWF2 protein abundance with spine length was observed (Fig. 8*E*), while the positive association between TWF2 and thin spine length trended toward significance (Fig. 8*F*).

**Figure 9.**
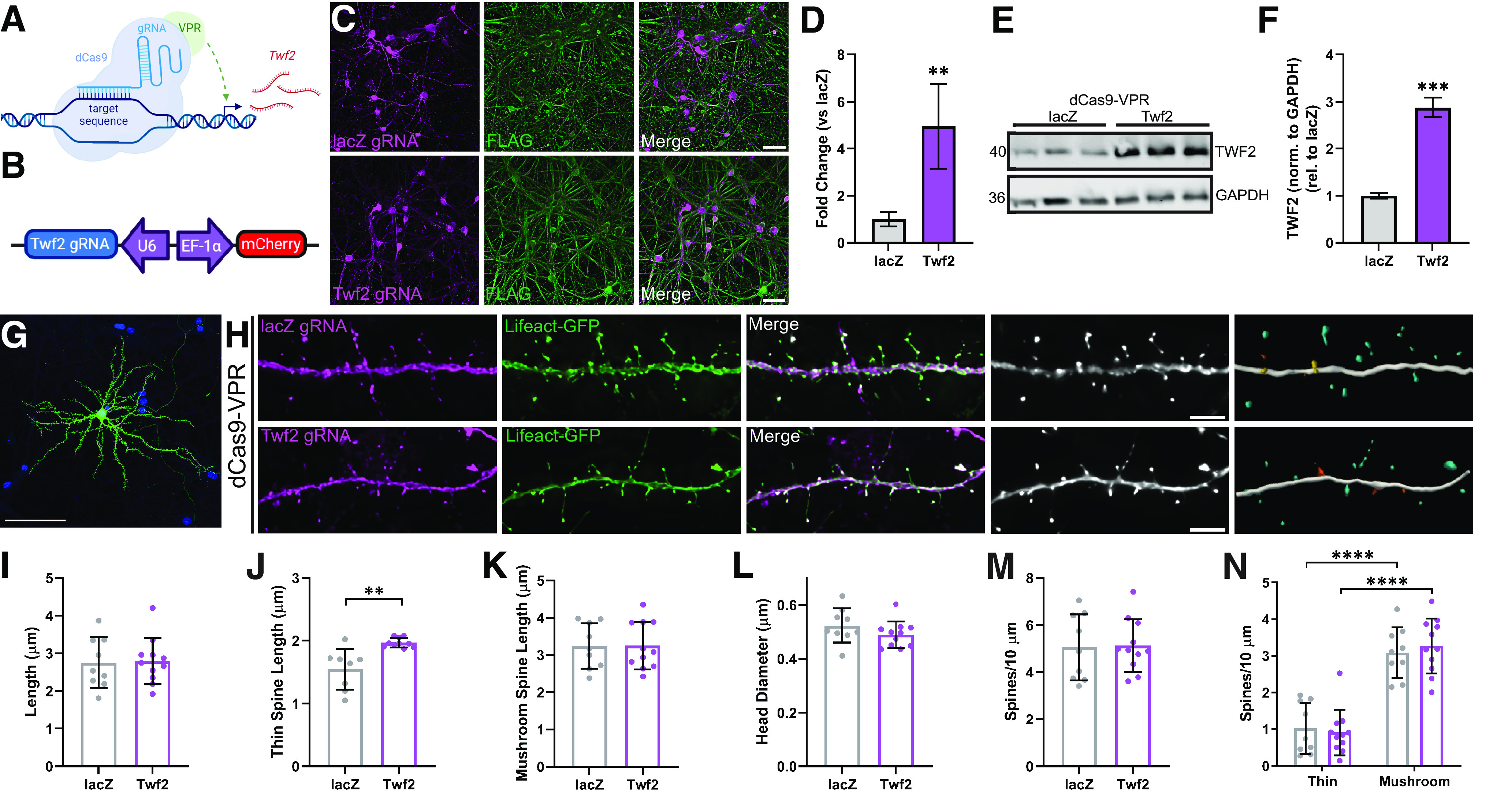
CRISPRa targeting Twf2 increases thin spine length. ***A***, Schematic of CRISPR activation (CRISPRa) targeting Twf2. CRISPR guide RNAs (gRNAs) target the transcriptional activator, dCas9-VPR, to the target sequence to upregulate transcription of the gene of interest, Twf2. ***B***, Schematic of the CRISPRa guide RNA used to target Twf2. The gRNA is expressed under the control of a U6 promoter. The construct co-expresses mCherry under an EF-1α promoter. ***C***, Representative images showing colocalization of dCas9-VPR (FLAG) and CRISPR gRNAs (mCherry) in DIV14 rat primary hippocampal neurons. Scale bar = 50 µm. ***D***, CRISPRa targeting Twf2 increases Twf2 mRNA 5-fold compared with lacZ. Unpaired *t* test (*t*_(6)_ = 4.320, *p* = 0.005). *N* = 4 lacZ and 4 Twf2. ***E***, Western blotting for TWF2 on rat primary hippocampal neuron lysates demonstrates increased TWF2 levels following CRISPRa targeting Twf2. ***J***, Quantification in ***F*** reveals that CRISPRa targeting Twf2 increases TWF2 protein 3-fold in rat primary hippocampal neurons, compared with lacZ nontargeting control. Unpaired *t* test (*t*_(4)_ = 14.57, *p* = 0.0001). *N* = 3 lacZ and 3 Twf2. ***G***, Representative image showing single hippocampal pyramidal neuron transfected with Lifeact-GFP. Nuclei in blue. Scale bar = 100 µm. ***H***, Representative images showing colocalization of lacZ or Twf2 gRNAs with Lifeact-GFP (images 1–3), as well as the deconvolved image (image 4) and 3D reconstruction (image 5). Scale bar = 5 µm. ***I***, Overall dendritic spine length was not altered by CRISPRa targeting Twf2 in rat primary hippocampal neurons, compared with lacZ control. ***J***, Elevating TWF2 abundance increased thin spine length in rat primary hippocampal neurons compared with CRISPRa targeting lacZ. Mann–Whitney *U* test (*U* = 7.0, *p* = 0.0019). *N* = 8 lacZ and 10 Twf2. ***K***, CRISPRa targeting Twf2 had no effect on mushroom spine length, in comparison to the lacZ nontargeting control. ***L***, Dendritic spine head diameter was comparable following CRISPRa targeting of lacZ and Twf2. ***M***, Dendritic spine density did not differ following CRISPRa targeting of Twf2 or lacZ. ***N***, Mushroom spine density is greater than thin spine density in rat primary hippocampal neuron cultures, with no effect of CRISPR manipulation on the density of each spine subclass. Two-way ANOVA (main effect of spine subclass: *F*_(1,36)_ = 101.4, *p* = 5.1 × 10^−12^) with Šídák's multiple comparisons test. lacZ ****p = 5.0 × 10^−6^, Twf2 ****p = 3.2 × 10^−9^. For ***I–N***, *N* = 9 lacZ and 11 Twf2. Each point represents one dendrite. Error bars represent the SD.

Based on the significant positive correlations between Module 16 and overall spine length and thin spine length, we predicted that increasing expression of the top hub protein, TWF2, would result in an increase in spine length. To test this hypothesis, we employed a CRISPRa system to increase endogenous TWF2 expression in rat primary hippocampal neurons (Fig. 9*A*). In this system, a catalytically dead Cas9 enzyme was fused to the transcriptional activator, VPR (VP64, p65, and Rta), referred to as dCas9-VPR. CRISPR guide RNAs (gRNAs) target dCas9-VPR to a specific genomic locus to drive upregulation of the gene of interest. gRNAs were driven by the U6 promoter and co-expressed mCherry for visualization (Fig. 9*B*). dCas9-VPR was driven by the human synapsin 1 promoter, enhancing neuron-specific transcription of the target gene. The dCas9-VPR construct also contained a FLAG tag for experimental visualization. The dCas9-VPR and gRNA constructs were packaged into separate lentiviruses (Savell et al., 2019).

Rat primary hippocampal neurons were transduced on DIV4 with two lentiviruses, one expressing dCas9-VPR, and the other expressing a gRNA targeting the rat *Twf2* gene. A gRNA targeting the bacterial gene *lacZ* was used as a nontargeting control for all CRISPRa experiments. On DIV14 neurons were fixed, immunostained for FLAG, and imaged to assess co-transduction of dCas9-VPR with each gRNA. Colocalization of FLAG and mCherry confirmed that dCas9-VPR and the *Twf2* or *lacZ* gRNAs were present within the same neurons (Fig. 9*C*). Additionally, RNA was isolated from neuronal cultures on DIV14 to quantify *Twf2* mRNA transcripts by RT-qPCR. We confirmed that CRISPRa targeting *Twf2* significantly increased *Twf2* mRNA 5-fold compared with the CRISPRa nontargeting control (Fig. 9*D*). To ensure that the increase in *Twf2* transcription resulted in greater TWF2 protein abundance, neurons were transduced on DIV4 and harvested on DIV14 for Western blotting and densitometry analysis. We observed that CRISPRa targeting *Twf2* increased TWF2 protein levels 3-fold compared with the *lacZ* nontargeting control (Fig. 9*E*,*F*).

To test whether upregulation of *Twf2* would increase overall spine length and thin spine length, rat primary hippocampal neurons were cultured on glass coverslips and co-transfected with the following three plasmids on DIV12: (1) dCas9-VPR; (2) *Twf2* gRNA, or *lacZ* control; and (3) Lifeact-GFP to visualize the actin cytoskeleton (Riedl et al., 2008). On DIV14, coverslips were fixed and imaged on a widefield microscope (Fig. 9*G*). Z-stacks of dendrites containing Lifeact-GFP and mCherry were captured at 60×. The z-stacks were deconvolved and digitally reconstructed in three dimensions to obtain measurements of dendritic spine density and morphology (Fig. 9*H*). While overall dendritic spine length was comparable in neurons following CRISPRa targeting of either *Twf2* or the *lacZ* control (Fig. 9*I*), thin spine length was significantly increased following upregulation of *Twf2* (Fig. 9*J*). However, mushroom spine length, which was not correlated with Module 16 eigenprotein expression, was not changed by CRISPRa targeting *Twf2* (Fig. 9*K*). Furthermore, other noncorrelated spine traits from the network, including head diameter and density, were not altered following *Twf2* upregulation (Fig. 9*L*,*M*). Rat primary hippocampal neurons contained significantly more mushroom spines than thin spines (Fig. 9*N*), whereas thin spines were more abundant on human EC dendrites (Fig. 6*D*). The difference in prevalence of spine subclasses between humans and rat neuron cultures likely prevented us from observing an effect of *Twf2* upregulation on overall spine length in cultured neurons.

Collectively, these findings suggest that TWF2 regulates length of thin dendritic spines, presumably through TWF2's action on actin cytoskeleton reorganization. Additionally, these results demonstrate that integration of dendritic spine measurements into a synaptic proteomic network enables unbiased identification of proteins that regulate specific spine traits. Furthermore, the correlational exclusivity of Module 16 with human spine length carried over into a relevant experimental model system following evaluation of the top module hub protein, indicating the specificity of this approach.

## Discussion

Proteomics is a powerful tool to capture disease-associated changes in protein expression. Correlation network approaches such as WGCNA have been used to cluster the proteome into biologically relevant modules of proteins to identify pathways and processes that are impacted in disease (Cerami et al., 2010; Tran et al., 2011; Clarke et al., 2013; Seyfried et al., 2017; Yu et al., 2018; Zhang et al., 2018; Johnson et al., 2020). In addition to identifying broad cellular functions that may be affected by disease, correlation network approaches have also been used to nominate individual proteins as drug targets or biomarkers (Huan et al., 2013; He et al., 2017; Sun et al., 2017; Toledo et al., 2017; Yue et al., 2017). However, few (if any) of such studies demonstrate functional validation of target proteins.

Dendritic spines are actin-rich protrusions on dendrites that are the postsynaptic site of the majority of excitatory synapses. TWF2 was identified as the hub protein of Module 16, which was exclusively correlated with dendritic spine length and thin spine length. TWF1 and TWF2 comprise the twinfilin family of actin binding proteins and have 65% sequence homology (Vartiainen et al., 2003). TWF1 regulates capping of actin filaments and sequesters actin monomers, which may promote actin filament depolymerization (Helfer et al., 2006; Johnston et al., 2015; Hakala et al., 2021; Mwangangi et al., 2021). TWF1 also modulates postsynaptic dendritic spine actin remodeling associated with opioid withdrawal, demonstrating a role for TWF1 at the synapse (Y.J. Wang et al., 2021). Similarly, TWF2 binds and sequesters actin monomers and regulates capping of actin filaments (Vartiainen et al., 2003; Nevalainen et al., 2009). TWF2 acts to limit elongation of shorter stereocilia in the cochlea, in conjunction with myosinVIIa (Peng et al., 2009; Rzadzinska et al., 2009). In contrast, TWF2 promotes the presence and length of neuronal processes on primary neurons and differentiated SH-SY5Y cells (Yamada et al., 2007), so the impact of TWF2 on actin filament growth and overall length of actin-based structures is not straightforward. Here, we showed that elevating endogenous TWF2 protein level by CRISPRa in rat primary hippocampal neurons increased thin spine length, without altering other noncorrelated spine traits. CRISPRa increased TWF2 by enhancing transcription of *Twf2* from the endogenous gene locus, which is in contrast to traditional overexpression approaches that ignore the endogenous gene locus and provide expression from an artificial, exogenous source (Savell and Day, 2017). These findings provide the first evidence to suggest that TWF2 can regulate actin cytoskeleton dynamics in dendritic spines. Further work will be required to determine the specific mechanism(s) of action, and whether this activity is specific to TWF2 or shared by TWF1 in neurons.

Our unbiased identification and functional validation of TWF2 from a cross-platform synaptic proteomic network demonstrates the value of integrating cellular information with proteomics. This framework represents a step forward in the use of proteomic data to identify and understand protein function in a biologically meaningful way. Both overall spine length and thin spine length significantly correlated with Module 16 eigenprotein expression in the network. However, we were only able to recapitulate the association with thin spine length in the functional validation studies. This is likely because of the difference in prevalence of spine types between humans and cultured rat neurons. Humans predominantly exhibit thin spines, while rodent primary neurons generate more mushroom spines (Boros et al., 2017, 2019; Henderson et al., 2019). Consideration of these differences between humans and model systems must be taken into account when functionally validating network targets. While not unexpected that an actin-binding protein affects spine morphology (Crick, 1982), the observation that the noncorrelated spine traits, including spine density and head diameter, were unaffected by CRISPRa targeting *Twf2* highlights the remarkable specificity of our network approach. Especially considering that TWF2 protein abundance likely originated from multiple different cell sources in the mass spectrometry experiments. Whereas, CRISPRa-mediated increases in endogenous TWF2 protein level originated exclusively from neurons in the *in vitro* experiments. Correlations between TWF2 protein abundance and spine length and thin spine length would have likely been stronger in human studies if the abundance of TWF2 could be measured exclusively from neurons.

Cognitively normal individuals with AD pathology are, by definition, in preclinical stages of AD, yet in life they appear resilient to clinical manifestations of dementia (Driscoll and Troncoso, 2011). We hypothesize that such individuals exhibit cognitive resilience that confers the ability to maintain cognitive function despite the accumulation of AD-related pathologies. Large-scale studies provide evidence for resilience to AD pathology, including the Religious Orders Study and the companion Rush Memory and Aging Project. These studies showed that a third of individuals in their eighties are cognitively normal despite levels of amyloid-β (Aβ) plaques and neurofibrillary tangles (NFTs) that meet NIA-Reagan criteria for intermediate to high likelihood of AD (Bennett et al., 2006). The Baltimore Longitudinal Study of Aging, Honolulu-Asia Aging Study, 90+ Study, and the Medical Research Council Cognitive Function and Ageing Study reported similar disconnect among Aβ plaques, NFTs and cognitive function (Head et al., 2009; O'Brien et al., 2009; Savva et al., 2009; White, 2009). Collectively, these findings suggest that approximately one-third of patients at risk for AD dementia exhibit some measure of resilience to AD pathology. Cognitive impairment in AD is the result of synapse and dendritic spine loss in brain regions that are critical for memory processes. This is based on numerous reports demonstrating that synapse markers and/or spine loss correlates more strongly with cognitive impairment in AD than Aβ plaques and NFTs (DeKosky and Scheff, 1990; Terry et al., 1991; Boros et al., 2017). Therefore, the ability to maintain cognitive abilities in an environment of AD pathology must be linked to the preservation and maintenance of synapses and spines in resilient individuals (Walker and Herskowitz, 2021). We discovered that dendritic spine structural remodeling is a plausible mechanism to maintain synapses and drive cognitive resilience in cognitively normal patients with AD pathology (Boros et al., 2017, 2019). Increases in spine length were exclusive to resilient cases among layer 2/3 neurons of the DLPFC (Boros et al., 2017), and thin spines were the most critical spine subtype in the DLPFC (Boros et al., 2017) and EC herein. We hypothesize that increases in thin spine length are critical to protect spines and synapses from AD pathology in cognitively normal patients exhibiting resilience. This study identified TWF2 as a mediator of thin spine length with some exclusivity. Therefore, future studies could modulate TWF2 protein level in rodent models to address whether thin spine remodeling is a mechanism of synaptic resilience against AD pathology.

This study had several limitations that should be considered. The sample size was low when compared with other proteomic studies on AD (Yu et al., 2018; Bai et al., 2020; Johnson et al., 2020; H. Wang et al., 2020). However, studies with a similar per-group sample size were able to capture disease-associated changes that were also detected by proteomic studies with larger sample sizes (Seyfried et al., 2017; Johnson et al., 2018; Zhang et al., 2018; Carlyle et al., 2021). Another potential limitation of this proteomic dataset was the use of LFQ. While our examination of peptide coefficients of variation revealed minimal variability among internal standards, which indicates robust reproducibility among samples, recently developed approaches such as tandem mass tag mass spectrometry could yield deeper proteomes. The sample size of the CAD group was low in comparison to the controls and AD cases, and thus represents a major limitation of this study. Because of the small sample size, interpretation of results and assurances of exclusivity of specific phenotypes within the CAD group are more challenging to accept. Another caveat is that the control samples were younger than the CAD and AD cases. However, CAD cases had comparable spine density to controls, suggesting that the reduction in spine density in the AD cohort was not solely because of differences in age. This indicates that diagnostic status is a confounding variable in the observed inverse correlation of spine density with age. Another potential major caveat in this study is PMI. Specifically, PMI was significantly longer in CAD cases compared with controls and AD cases. Pearson correlations between dendritic spine measurements and PMI revealed statistically significant associations, including spine length and head diameter. While CAD cases did not exhibit statistically significant differences in spine length or head diameter in comparison to controls or AD patients, it is possible that the altered PMI of CAD cases masks differences among spine traits when compared with the control and AD groups. Notably, a study on various factors affecting postmortem human brain ultrastructure found that PMI did not significantly correlate with number of neuronal profiles, which is an indicator of overall tissue quality. However, the authors did observe a significant reduction in number of postsynaptic densities in the prefrontal cortex with increasing PMI (Glausier et al., 2019).

In summary, we demonstrate that integrating dendritic spine measurements with a synaptosome proteome co-expression network enabled the unbiased identification of TWF2 as a potential regulator of thin dendritic spine length. This approach of incorporating relevant cellular traits with proteomic data could better facilitate the selection of proteins to elucidate physiological and pathologic processes, as well as drug targets or biomarkers. Further work is necessary to determine whether this framework is applicable and translatable to other cellular traits or specific disease metrics.
